# Transcriptome profiling disclosed the effect of single and combined drought and heat stress on reprogramming of genes expression in barley flag leaf

**DOI:** 10.3389/fpls.2022.1096685

**Published:** 2023-01-16

**Authors:** Krzysztof Mikołajczak, Anetta Kuczyńska, Paweł Krajewski, Michał Kempa, Maria Nuc

**Affiliations:** Institute of Plant Genetics, Polish Academy of Sciences, Poznań, Poland

**Keywords:** abiotic stress, combinatorial stresses, flag leaf, functional annotation, *Hordeum vulgare* L., mRNA sequencing, transcriptomics

## Abstract

Despite numerous studies aimed at unraveling the genetic background of barley’s response to abiotic stress, the modulation of the transcriptome induced by combinatorial drought and increased temperature remains largely unrecognized. Very limited studies were done, especially on the flag leaf, which plays an important role in grain filling in cereals. In the present study, transcriptome profiles, along with chlorophyll fluorescence parameters and yield components, were compared between barley genotypes with different flag leaf sizes under single and combined drought and heat stress. High-throughput mRNA sequencing revealed 2,457 differentially expressed genes, which were functionally interpreted using Gene Ontology term enrichment analysis. The transcriptomic signature under double stress was more similar to effects caused by drought than by elevated temperature; it was also manifested at phenotypic and chlorophyll fluorescence levels. Both common and stress-specific changes in transcript abundance were identified. Genes regulated commonly across stress treatments, determining universal stress responses, were associated, among others, with responses to drought, heat, and oxidative stress. In addition, changes specific to the size of the flag leaf blade were found. Our study allowed us to identify sets of genes assigned to various processes underlying the response to drought and heat, including photosynthesis, the abscisic acid pathway, and lipid transport. Genes encoding LEA proteins, including dehydrins and heat shock proteins, were especially induced by stress treatments. Some association between genetic composition and flag leaf size was confirmed. However, there was no general coincidence between SNP polymorphism of genotypes and differential expression of genes induced by stress factors. This research provided novel insight into the molecular mechanisms of barley flag leaf that determine drought and heat response, as well as their co-occurrence.

## Introduction

Today, it is known that understanding the transcriptome is important for interpreting the functional elements of the genome. Sustainable improvement of genetic and molecular techniques has facilitated a better exploration of plant genomes, including barley (Harb et al., 2020; Henry, 2020). Next generation sequencing (NGS), providing high-throughput analyses at the genome-wide level in a cost-effective manner, allowed us to unlock the barley genome (cv. Morex) in 2012, for the first time (IBGSC, 2012). In the following years, updated versions of the reference barley genome were published by Mascher et al. (2017) using chromosome conformation capture mapping and by Monat et al. (2019), who employed short-read sequencing data from multiple library types (TRITEX). Very recently, the newest version of barley genome assembly (MorexV3) was released using PacBio long-read sequencing (Mascher et al., 2021). Also, the first barley pan-genome concept was reported by Jayakodi et al. (2020), whereas Rapazote-Flores et al. (2019) released the barley gene reference transcript dataset (BaRTv1.0). Altogether, powerful resources for extensive research into the barley genome and its functioning under changing environmental conditions have been made available.

Climate extremes, like dryness and heat, have intensified in recent years due to global warming. The Intergovernmental Panel on Climate Change (a United Nations body) projected that warming of the Earth’s atmosphere by every 0.5°C would result in more frequent and omnipresent drought episodes with a noticeable increase in their severity around the globe (the IPCC, 2021 report). Plants permanently struggle for survival under various environmental stressors and in field conditions, they are usually exposed to several hazards rather than just one (Suzuki et al., 2014). For instance, drought is often accompanied by high temperatures, and the molecular response of plants to combined stress cannot be easily deduced from the effect of each stress alone (Prasad et al., 2011). Some genes, i.e., those determining the trade-off between signaling pathways (e.g., phytohormones) or the underlying universal stress response (e.g., detoxification), can have the same regulatory status under different stresses (Prasch and Sonnewald, 2015). On the other hand, two different stress factors may induce a stress-specific strategy, sometimes requiring an opposite response from the plant. For instance, opening and closing of stomata are preferred by plants during exposure to heat and drought, respectively (Zandalinas et al., 2016; Hsu et al., 2021). Therefore, during their co-occurrence, the plant may generate a unique molecular response, too. The literature about the combined effects of abiotic stresses on barley is quite limited and mainly based on the physiological comparison of barley genotypes rather than the changes in gene expression profiles (Ahmed et al., 2013). Recognition of barley transcriptome modulation induced by combinatorial abiotic stresses, including drought and increased temperature, is still largely missing.

Response of plants to constrained conditions is a very complex phenomenon and involves reactions of a multitude of mechanisms. To date, many studies have focused on plants’ molecular adaptation to environmental stresses, and numerous stress-induced genes have been reported, also in barley (Gürel et al., 2016; Zhao et al., 2020; Mareri et al., 2022). This includes the examination of the protective role of late embryogenesis-related proteins (LEA) during dehydration, and dehydrins belonging to group 2 of LEA are the most commonly studied in various plant species (Yu et al., 2018). Additionally, genes encoding group 3 of LEA proteins have been widely described as responsive to drought, salinity, heat, and cold stresses (Xiao et al., 2007; Kosová et al., 2014). Also, the regulatory network of abscisic acid (ABA), a prominent messenger of plant stress response, has been extensively studied (Ma et al., 2018); candidate genes for ABA biosynthesis and catabolism in barley have been proposed (Fidler et al., 2015). A large number of genes with potential roles in heat stress responses have been identified, with the central role being played by those encoding heat shock proteins (HSPs) (Chang et al., 2007). They play a role in the protection of intracellular proteins from denaturation and preserve their stability and function through protein folding; thus, they act as chaperones (Baniwal et al., 2004). Different HSP-encoding genes have been listed in barley (Reddy et al., 2014). A crucial role of heat stress factors (HSFs) in the induction of the heat stress response has also been confirmed in model plant studies, since they regulate thermal protection through interaction with HSPs (Scharf et al., 2012). Candidate genes encoding HSFs have been proposed in barley by phylogenetic analysis (Mishra et al., 2020).

Despite the large efforts made to study the complex regulatory mechanisms underlying plant response to stress factors, the genetic and environmental regulation of plant adaptations, including barley, to various conditions (e.g., drought, heat), remains largely unrecognized (dos Santos et al., 2022). Although the importance of the flag leaf in flowering and grain yield determination has been studied, relatively little research has been done on the molecular characterization of the flag leaf of barley. Abiotic stresses occurring at the reproductive stage limit the availability of nutrients translocated from photosynthetic and storage organs. At this developmental stage, the viability of the flag leaf and its efficient photosynthesis activity are of great importance (Verma et al., 2004). The flag leaf plays a fundamental role in grain filling in cereals. As the top-most leaf, it intercepts quite a lot of sunlight and feeds the spike by translocation of assimilates. When the flag leaf is lost or destroyed, grain yield is reduced (Ojanpera et al., 1992). Our previous studies demonstrated that a terminal water shortage imposed in the flag leaf stage was more devastating for yield components compared to the early stress in barley (Mikołajczak et al., 2017). Undoubtedly, profound molecular studies are needed to fill the gap about the flag leaf’s behavior under environmental stimuli. Most transcriptomics reports focus on younger leaf examination (e.g., Wehner et al., 2016; Zeng et al., 2016), less on roots (e.g., Janiak et al., 2019), and very limited studies about genome-wide expression changes in barley crown tissue have been reported (Svoboda et al., 2016; Mikołajczak et al., 2022). The sparse evidence corresponding to molecular characteristics of barley flag leaf is related mainly to metabolite profiling (Templer et al., 2017; Niu et al., 2022). Also, Hosseini et al. (2016) examined the effect of potassium treatment on primary metabolism and abscisic acid accumulation in barley flag leaf of two genotypes contrasted in stress tolerance and exposed to temporal drought. Additionally, the authors analyzed the expression changes of two ABA-related genes *via* real-time qPCR. In fact, the global transcriptome re-modeling in flag leaf, especially under combined drought and heat, has not been investigated in barley so far.

Hence, this study was aimed at deciphering the impact of drought and heat on genome-wide gene expression in the flag leaf of barley. We employed high-throughput sequencing of mRNA to identify genes that are associated with responses to drought or heat and their combinations. We assumed that changes in the transcriptome under a combined stress cannot be considered as the additive effects of the single stresses, however some subset of genes, e.g., with universal function, may react identically in different environments. Secondly, we hypothesized that stress-induced changes in gene expression depend also on the morphology of the flag leaf. However, we expected that although the morphology of the flag leaf is genetically determined, single nucleotide mutations do not have an unambiguous effect on the different expression of polymorphic genes under unfavorable conditions. Transcriptome data was analyzed along with the evaluation of phenotypes and chlorophyll fluorescence parameters of the flag leaf.

## Material and methods

**Figure 1:**
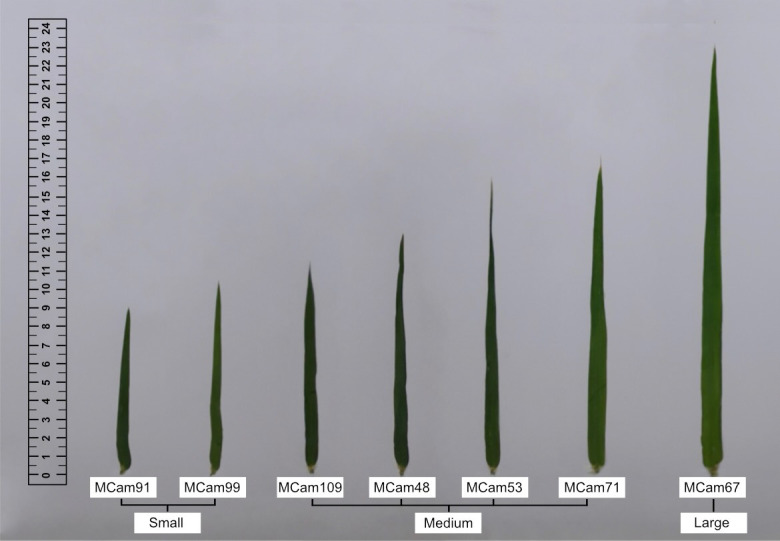
Flag leaves of seven barley accessions (RILs) classified into three groups according to flag leaf size: small, medium, and large.

Plant material consisted of seven recombinant inbred lines (RILs, called MCam) of spring barley developed by crossing the cv. Maresi (European) and CamB1 (Syrian) breeding lines (Mikołajczak et al., 2016; Mikołajczak et al., 2017; Mikołajczak et al., 2020) in the Team of Cereal Phenomics, Institute of Plant Genetics, Polish Academy of Sciences. Accessions were classified into three groups according to flag leaf size: small (S), medium (M), and large (L) (**Table 1**, **Figure 1**). Five flag leaves of each genotype were used to define the average dimensions and calculate the leaf area (of the rectangle framing the leaf).

**Table 1:** Grouping of barley genotypes with respect to the flag leaf size.

Genotype	Flag leaf dimensions (cm)				Area (rect., cm^2^)	Rank	Group according to flag leaf size
	Length	s.e.m.	Width	s.e.m.			
MCam48	10.0	0.3536	0.84	0.02449	8.4	4	medium
MCam53	13.4	0.967	0.86	0.04	11.524	5	medium
MCam67	22.2	0.5148	0.98	0.03742	21.756	7	large
MCam71	16.3	0.7176	0.84	0.06	13.692	6	medium
MCam91	6.0	0.3536	0.62	0.03742	3.72	1	small
MCam99	7.8	0.3391	0.54	0.02449	4.212	2	small
MCam109	7.9	0.4	0.76	0.04	6.004	3	medium

### Abiotic stress application

Experiment was carried out in phytotrons under fully controlled conditions according to Kuczyńska et al. (2019) and Mikołajczak et al. (2022) with modifications. The 60%–70% of air humidity and 234 µmol m^−2^ s^−1^ PAR irradiance (Apollo 8 LED Grow Light) were applied. Each pot (H-LSR 4.5 L; 21 cm in diameter and 20 cm in height) was filled with a mixture of loamy soil and peat (3:1, *w*/*w*), then pots were arranged randomly on the phytotron benches. Ten seeds were sown in each pot, and the number of plants was reduced to five after germination. Firstly, all plants were grown at 8°/14° C and 12/12 h (night/day) photoperiod for one month and next at 16°/22° C and 8/16 h (night/day) photoperiod until the end of the vegetation season under optimal soil moisture, i.e., at above 70% field water capacity (FWC), with some changes in stressed variants. Overall, four environmental variants were applied with two temperature and water regimes: control conditions (C) as described above; drought (D)—soil moisture at 20% FWC and temperature as in C; high temperature (H)—20°/30° C (night/day) and optimal soil moisture; and combination of the stress treatments (HD)—20°/30° C (night/day) and 20% FWC. Abiotic stress was imposed at the flag leaf stage (39 BBCH, approx. 29–47 days after sowing depending on the genotype) and maintained for seven days. Biological samples (flag leaves) for molecular analyses were collected at two time points, i.e., on the third (T1) and seventh (T2) days of stress. Soil moisture was controlled gravimetrically by the daily weighing of each pot. The number of pots was set to provide sufficient material for molecular studies and phenotyping.

### Whole-genome expression analysis

Flag leaves of barley genotypes were collected from all experimental variants at two time points (T1 and T2), and they were immediately frozen in liquid nitrogen and stored at −80°C until analysis. Total RNA (1–2 µg) was extracted in triplicates using the Qiagen (RRID : SCR_008539, http://www.qiagen.com, Hilden, Germany) RNeasy Plant Mini Kit according to the manufacturer’s instructions. Genomic DNA contamination was removed twice, i.e., on-column DNase digestion (RNase-Free DNase Set, Qiagen) and using the DNase Max Kit (Qiagen) during and after RNA isolation, respectively. Three flag leaves sampled from different plants of each genotype in a pot formed one replication, and three such replications were examined. RNA quantity, quality, and integrity were evaluated following the study by Mikołajczak et al. (2022). The construction of a cDNA library (TruSeq stranded mRNA) and mRNA sequencing were performed by Macrogen Inc. (RRID : SCR_014454, http://www.macrogen.com, Seoul, Republic of Korea) using an Illumina NovaSeq6000 platform with a 150-bp paired-end configuration and the number of read pairs ranging from 18.3 to 40.9 M per sample.

### Phenotype characteristics

All plants were manually harvested after ripening (approximately 69–84 days after sowing depending on the genotype and treatment), and plant architecture-related traits along with yield components were measured. Both primary (main) and secondary (lateral) stems were examined, as well as the main phenological stages were noted; overall, 26 characteristics were evaluated (T1–T26). Additionally, nine chlorophyll fluorescence parameters (F1–F9) were measured using the FluorPen FP 110/D device (Photon System Instruments, PSI, Drásov, Czech Republic) on the flag leaf of plants at the third (T1) and seventh (T2) days of stress across treatments. After a 30-minute dark adaptation, employing detached leaf-clips, flag leaves were immediately exposed to a pulse of saturating light at an intensity of 3,000 μmol m^−2^ s^−1^ and all parameters were measured. Three replicates were used for the above-mentioned observations, each consisting of five plants from one pot. Full list of analyzed phenotypic traits and chlorophyll fluorescence parameters, with corresponding identifier/abbreviation, are given in **Supplementary Table 9A** and **Supplementary Table 9B**, respectively.

### Data analysis

The IBSC_v2 *Hordeum vulgare* (Ensembl Plants rel. 48; RRID : SCR_008680, http://plants.ensembl.org/index.html) genome assembly was used as a reference for SNP and gene expression analyses. After removing adapter-related sequences and quality trimming using AdapterRemoval ver. 2.1.7 (RRID : SCR_011834, https://github.com/MikkelSchubert/adapterremoval, Schubert et al., 2016) (parameters: –minquality 20, –minlength 50), mRNA-seq reads were mapped into hi the reference sequence using TopHat ver. 2.1.1 (RRID : SCR_013035, http://ccb.jhu.edu/software/tophat/index.shtml, Kim et al., 2013) (parameters: maximum no. of mismatches = 1, –no-mixed, –no-discordant); the mapping efficiency was 61.8%–83.9%. Reads aligned to transcripts were counted using the featureCounts function in Bioconductor (RRID : SCR_006442, http://www.bioconductor.org/), R 3.6.1 (RRID: SCR_001905, http://www.r-project.org/, Rsubread library; Liao et al., 2019), and the resulting data were subjected to differential expression analysis in DESeq2 ver. 1.22.2 (RRID: SCR_015687, https://bioconductor.org/packages/release/bioc/html/DESeq2.html) (Love et al., 2014). Differentially expressed genes (DEGs) between treated and control samples were found among the genes characterized by a mean expression of at least 10 units (estimated in DESeq2), |log_2_(FC| >3, and FDR <0.01. Principal coordinate analysis (PCoA) on log_2_(FC) values for DEGs was based on the matrix of Euclidean distances. Gene Ontology term enrichment analysis was performed using GO Slim annotation and tools at geneontology.org (RRID : SCR_002811). A weighted gene co-reaction network analysis was performed using the WGCNA library (RRID : SCR_003302, http://www.genetics.ucla.edu/labs/horvath/CoexpressionNetwork/) in R (Langfelder and Horvath, 2008; Langfelder and Horvath, 2012) with the following parameters: beta = 10, complete link clustering method, cutHeight = 0.95, minsize = 20. SNP calling in mRNA-seq data pooled from three biological replications separately for seven genotypes, in optimal conditions at T1, was performed using the samtools/bcftools pipeline (Li, 2011) (filtering parameters: %QUAL >60, MAF >0.10, DP >80). Venn diagrams were drawn using the “venn” package in R. SNP protein translation effects were predicted using the VEP tool (Ensembl Plants; McLaren et al., 2010). SNP data were analyzed using PCoA based on a matrix of simple matching similarity coefficients between genotypes and hierarchical clustering by complete link method. Statistical tests and analyses of phenotypic and chlorophyll fluorescence data were performed in GenStat 19 (RRID : SCR_014595, http://www.vsni.co.uk/products/genstat/) (VSN International, 2017). Principal component biplots were created after centering and normalizing the data. An analysis of variance was performed in a model containing fixed effects for treatment, flag leaf size group, time point, and the interactions of pairs these factors. A weighted trait correlation network analysis performed jointly for contrasts on phenotypic and expression data using the WGCNA library with the following parameters: beta = 6, complete link clustering method, cutHeight = 0.98, minsize = 10.

## Results

### Whole-genome expression analysis

**Figure 2:**
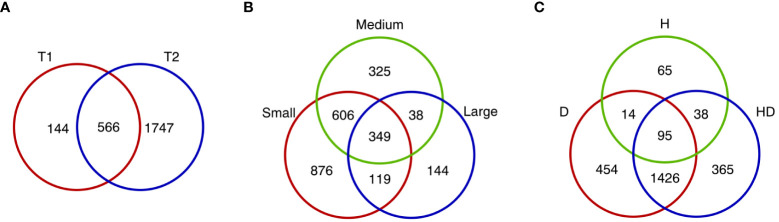
Venn diagrams visualizing the number of differentially expressed genes in flag leaves, specific and shared between: **(A)** time points (T1 and T2), **(B)** barley genotypes assigned to groups of different sizes of flag leaf (small, medium, and large), and **(C)** applied stress treatments (D, H, and HD).

Overall, 2,457 differentially expressed genes (DEGs) were detected in at least one of the 18 comparisons between the three treatments (H, D, and HD) and control conditions (C) for three flag leaf size groups at two time points (**Supplementary Table 1**). In general, accessions with a small size of the flag leaf (S) had a greater number of downregulated genes (with the exception of T1 and D), whereas in the large leaf group (L), upregulated genes were more numerous, independently of the type of stress and time of sampling; plants with a medium flag leaf size (M) had more upregulated genes in D and HD but less in H than downregulated ones at both time points. A larger number of genes showed induced expression in response to D and HD than to H, especially at T2 (**Table 2**). About 3-fold more DEGs were observed at the seventh day of stress (T2) than on the third day (T1) (**Figure 2A**), however in group M the number of DEGs in H treatment was similar at both time points (**Table 2**). Most of the DEGs at T1 (approx. 80%) were also present at T2. Genotypes in group S had 2-fold more common DEGs with plants in group M than with those in group L; significantly, 349 DEGs were shared between all classified groups (**Figure 2B**). There were 1,521 DEGs shared between D and HD, whereas 95 DEGs were shared across all stress conditions. Treatments D and HD affected the expression of ca. 9-fold more genes than H (**Figure 2C**).

**Table 2:** Number of down- and upregulated genes in genotypes classified to different groups according to flag leaf size, at time points T1 and T2, in comparisons between drought (D), heat (H), their combination (HD) v. control.

Group according to flag leaf size	Regulation status	T1			T2		
		D	H	HD	D	H	HD
Small	down	29	5	93	855	45	844
	up	47	2	41	767	22	614
	total	76	7	134	1622	67	1458
Medium	down	48	59	173	102	62	271
	up	290	21	281	727	18	613
	total	338	80	454	829	80	884
Large	down	0	4	3	182	11	222
	up	16	11	25	208	12	242
	total	16	15	28	390	23	464

Principal coordinate analysis (PCoA) of log_2_(FC) values for DEGs showed a lower variability in reaction of gene expression to applied stresses in flag leaves of group L than in group M, and transcriptomic reaction in group S was substantially different from the others (**Supplementary Figure 1**).

For the functional interpretation of differentially expressed genes, the Gene Ontology (GO Slim, geneontology.org) enrichment analysis was done for a set of DEGs significant in at least three contrasts (1,037 genes) (**Supplementary Table 2**). Mainly, overrepresented GO terms were related to “photosynthesis” and “response” to various factors; the term “response to abiotic stimulus” was the most significantly enriched in all cases (except for group L). Also, the term “response to abscisic acid” was significantly enriched in all cases (except for the H treatment). Within the groups defined by flag leaf size, the DEGs identified for group L determined the lowest number of enriched GO terms. A lower number of enriched GO terms was observed for H than for D and HD. However, GO terms shared across all treatments were more significant in H than in other treatments, interestingly, including “response to osmotic stress” and “response to oxidative stress.” In general, a set of enriched GO terms did not change over time (they were slightly more numerous in T2), and similar significance for each of them was observed between time points. A few enriched GO terms were specific to flag leaf size, stress variant, or time point (**Supplementary Table 2**). It is worth not that terms “response to heat,” “response to oxidative stress,” and “response to osmotic stress” were overrepresented in leaves of group M, whereas terms associated with phosphatase activity and dephosphorylation were specific to the second time point (T2).

Secondly, to interpret functionally a set of genes whose expression responded similarly to stress, we used log_2_(FC) values for 18 contrasts to construct the gene co-reaction network, which revealed 15 modules (M1–M15) (**Supplementary Table 1**, **Supplementary Table 3**). For a better summarization, the obtained modules were further grouped according to the similar response of DEGs assigned to each module (based on module eigengenes). On this basis, we selected three joined modules (MI–MIII) containing DEGs with the most similar expression (**Table 3A**), for which the GO Slim enrichment analysis was performed (**Table 3B**). In general, DEGs in module MI (86 genes) reacted positively in H relative to control condition C, whereas in D and HD gene expression was reduced or similar to that of condition C. These DEGs were numerously annotated as oxidoreductases, receptor-like protein kinase, and chlorophyll a/b binding proteins (according to the NR protein database). These findings were interrelated with GO term enrichment results, which revealed, i.e., that the terms photosynthesis and response to abiotic or to light stimuli were overrepresented in this set of DEGs.

**Table 3A:** Joined co-reaction modules containing DEGs with the most similar expression reaction induced by stress.

Group according to flag leaf size	Time point	Treatment	MI	MII	MIII
S	T1	D	0.70	−0.17	0.41
M	T1	D	0.00	0.51	0.49
L	T1	D	0.38	−0.20	0.35
S	T2	D	−1.72	1.25	1.11
M	T2	D	−0.27	1.51	1.07
L	T2	D	−0.69	0.46	0.58
S	T1	H	0.84	−0.88	−0.37
M	T1	H	0.70	−1.05	−1.64
L	T1	H	0.91	−1.02	−1.16
S	T2	H	0.78	−0.92	−1.13
M	T2	H	0.94	−0.87	−1.31
L	T2	H	0.81	−0.94	−1.04
S	T1	HD	0.22	−0.46	0.17
M	T1	HD	−0.55	0.34	0.19
L	T1	HD	0.35	−0.44	0.00
S	T2	HD	−1.89	1.05	0.88
M	T2	HD	−0.83	1.36	0.96
L	T2	HD	−0.67	0.47	0.45

**Table 3B:** GO Slim terms overrepresentation analysis of three joined co-reaction modules.

GO term|GO identifier	Overrepresentation score (−log_10_(q-value))		
	MI	MII	MIII
cellular lipid catabolic process|GO:0044242	–	–	1.88
generation of precursor metabolites and energy|GO:0006091	5.03	–	–
lipid catabolic process|GO:0016042	–	–	2.18
photosynthesis|GO:0015979	9.23	–	–
response to abiotic stimulus|GO:0009628	5.83	−	−
response to abscisic acid|GO:0009737	−	2.13	−
response to cold|GO:0009409	−	4.39	−
response to inorganic substance|GO:0010035	−	2.43	−
response to light stimulus|GO:0009416	7.63	−	−
response to lipid|GO:0033993	–	1.82	−
response to radiation|GO:0009314	7.56	−	−
response to stimulus|GO:0050896	1.87	−	−
response to temperature stimulus|GO:0009266	−	1.95	−

DEGs assigned to module MII (225 genes) were strongly expressed in D and HD relative to C and H independently from the type of flag leaf in T2; it was also confirmed in T1, but only for genotypes belonging to group M. This module was, again, rich in oxidoreductase-related genes (NR protein database) and numerous hormone-related genes were observed, mainly corresponding to the abscisic acid regulatory pathway (Plant Reactome). We confirmed that the GO term “response to abscisic acid” was overrepresented within DEGs of module MII. Among the enriched GO terms we also found, i.e., the terms “response to cold” and “response to temperature stimulus.”

Expression profiles of genes from module MIII (55 genes) were similar to those from module MII for all groups of flag leaf size over time, except for DEGs affected by D and HD in T1, which had rather positive regulation status compared to module MII in groups S and L. Genes associated with ABA biosynthesis and signal transduction and with the MAPK signaling pathway were particularly abundant in module MIII. However, only terms related to lipid catabolism were found to be overrepresented.

Next, GO term enrichment analysis (at geneontology.org) was applied to uncover functions of 349 DEGs common between all groups defined by flag leaf size (**Figure 2B**, **Supplementary Table 4A**) and 95 DEGs shared across stress treatments (**Figure 2C**, **Supplementary Table 4B**). In the first case, the biological process category contained the most enriched terms, mainly associated with responses to various factors, including water deprivation, temperature stimulus, and abscisic acid; the term “cold acclimation” had the highest fold enrichment. Three overrepresented GO terms were detected within the molecular function category (the term “carotenoid dioxygenase activity” enriched the strongest), but none of these terms was found for a cellular component. DEGs assigned to these GO terms were present in all modules defined by the gene co-reaction network (except for M4 and M7). For the set of DEGs shared between treatments (belonging to modules M6, M7, M9–M13), enriched GO terms were mostly similar to those mentioned above within the biological process category; additionally, “response to oxidative stress,” “response to ROS,” and some photosynthesis-related GO terms were overrepresented. Within the molecular function category, there were, among others, the terms “gibberellin 20-oxidase activity” and “binding of protein, tetrapyrrole, or chlorophyll”. In general, terms associated with photosystems I and II as well as chloroplasts were enriched in the cellular component category.

Lastly, 365 genes whose expression was exclusively affected by combined drought and heat were used for GO term overrepresentation analysis. However, no significant results were found within the biological process, molecular function, and cellular component categories. Therefore, we additionally tested Panther Protein Class overrepresentation (at pantherdb.org) and discovered a few enriched enzyme-related terms, i.e., “oxygenase,” “oxidoreductase,” “metabolite interconversion enzyme,” “hydrolase,” and the term “transporter” (**Figure 2C**, **Supplementary Table 4C**).

#### Subset of DEGs assigned to selected functional GO annotations

**Figure 3:**
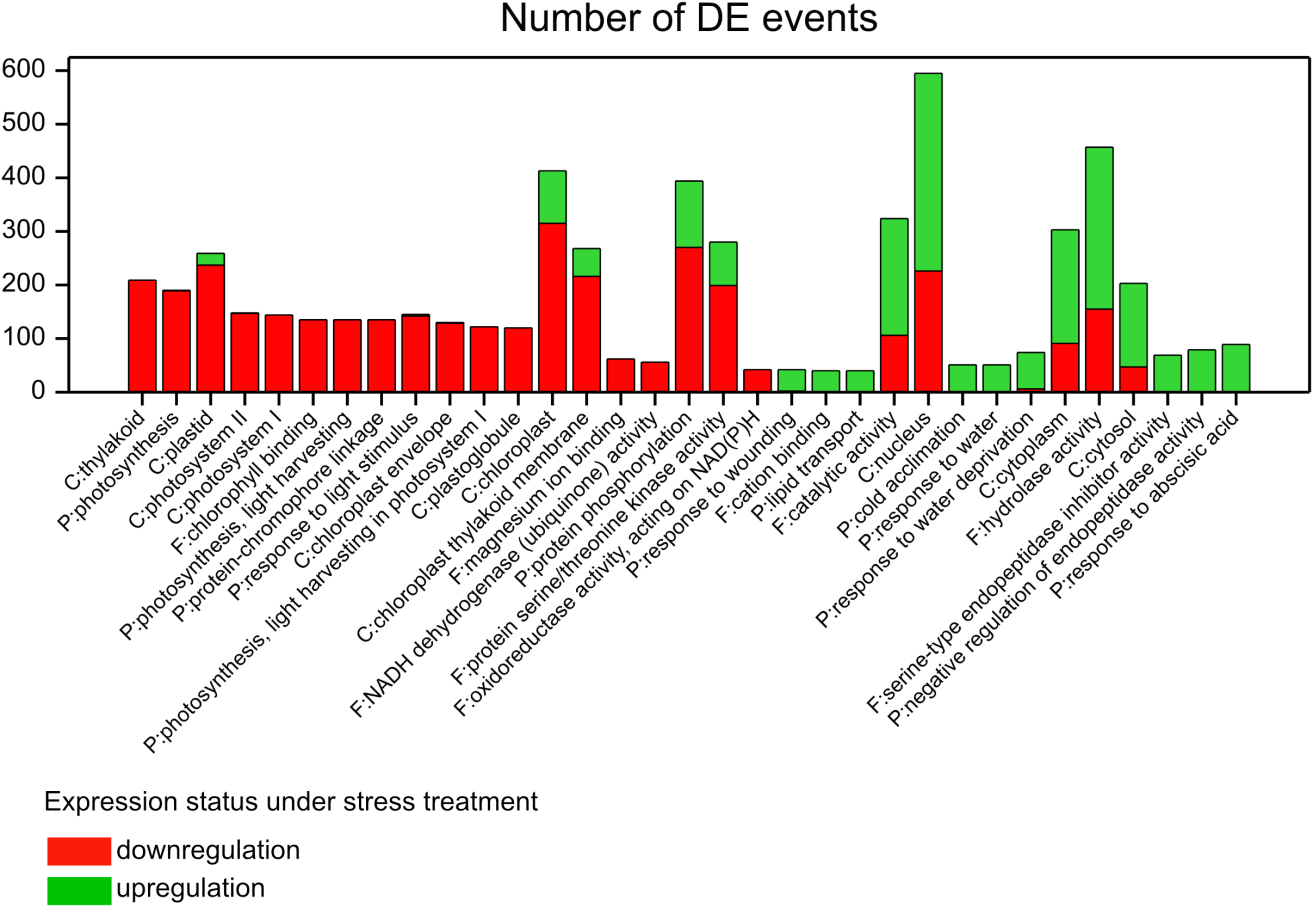
Enriched GO terms with assigned DE events whose distribution was significantly different than the marginal distribution of all DE events in the experiment (χ^2^ test, P <0.001, |std. residual| >6); C, cellular component; F, molecular function; P, biological process.

We extracted GO terms annotating sets of DEGs (see the GO annotation in **Supplementary Table 1**) within which the distribution of genes, with respect to direction of up and downregulation, was significantly different from the calculated marginal distribution of all differential regulation events in the whole experiment (3,008 down/3,957 up). This approach enabled us to visualize for which GO term the expression of assigned genes was particularly increased or reduced under applied treatments (**Figure 3**, **Supplementary Table 5**); Furthermore, in this analysis one gene could be considered in several “differential expression events” (DE events) and could show different expression reactions across defined contrasts.

##### DEGs assigned to photosynthesis and closely related terms

In general, DEGs associated with photosynthesis and most of the terms closely related to it reacted negatively to stress factors relative to control. In fact, there were 53 DEGs assigned to photosynthesis whose expression was reduced in response to stress (189 DE events), excluding HORVU2Hr1G090070, which was exclusively upregulated in genotypes of group S under D in T2 (**Supplementary Table 6**). Almost 90% of these DEGs were observed for group S, especially in T2 for D and HD (including 11 genes specific to HD), whereas in T1 only HD induced some significant changes in gene expression. About 40% of DEGs were also identified for group M, mostly in HD at both time points. Only one gene, HORVU2Hr1G010690, had reduced expression in group L in D and T2. About 1/4 of photosynthetic DEGs were shared across treatments but no common DEGs between H and HD, being parallelly not significant in D, were identified. Interestingly, 4-fold more DEGs induced by H were detected in T1 than in T2. Genes encoding chlorophyll a/b binding proteins were the most numerously represented in this subset of DEGs. Few genes were associated with the photosystem I reaction center subunit.

On the other hand, there were some DEGs assigned to the terms “chloroplast” and “chloroplast thylakoid membrane” (cellular components) whose expression reacted positively to stress; about 1/4 and 1/5 of all DE events were “upregulations,” respectively (**Figure 3**, **Supplementary Table 5**). More than half DEGs were shared between D and HD being not significant in H. Interestingly, there was one upregulated DEG, HORVU2Hr1G038830, common between H and HD (not present in D) in group L in both time points and in group M in T1; it encoded ferredoxin-NADP reductase (**Supplementary Table 6**). The expression profile of downregulated genes assigned to both terms was similar to the above-mentioned photosynthesis related DEGs since they were partially shared between the two GO terms. Additionally, we found downregulated DEGs associated with ATP synthase CF1 epsilon subunit (six genes) and with NADH-plastoquinone oxidoreductase subunits 3, J, and K (12 genes), common between “chloroplast” and “chloroplast thylakoid membrane” terms, whose expression was specific to group S (in T2) in D and in D and HD, respectively. In turn, enhanced expression of genes was observed mostly in groups S and M under D and HD, especially in T2. Among them four genes encoded high molecular mass early light-inducible protein HV58. Several upregulated genes encoded unknown proteins (predicted proteins according to the NR protein database); however, using the Plant Reactome database, we uncovered that HORVU4Hr1G050510 was involved in the HSFA7/HSFA6B-regulatory network induced by drought and ABA, whereas HORVU2Hr1G005320 was related to arginine and proline metabolism. Also, one of them, HORVU3Hr1G070850, encodes the malic enzyme (EC:1.1.1.40). Three DEGs were found to have opposite regulation status in group M, i.e., gene expression was enhanced in D and HD in T2 but reduced in T1 in H. They encoded putative 9-cis-epoxycarotenoid dioxygenases (HORVU5Hr1G044510, HORVU5Hr1G054970), and HORVU5Hr1G092850 (a predicted protein) was involved in ABA biosynthesis and mediated signaling according to the Plant Reactome database (**Supplementary Table 1**).

##### DEGs assigned to drought response

One of the GO terms revealed above corresponds to “response to water deprivation,” where 18 DEGs were assigned. Generally, they reacted positively in response to applied stress (**Figure 3**, **Supplementary Table 5**), mostly in D and HD in T2 in groups S and M, less in L; it was also confirmed for group M in T1 (**Supplementary Table 6**). Three DEGs were specific to D, one to H, and none to HD. The gene HORVU7Hr1G088140 encoding abscisic acid receptor PYL2 had reduced expression in HD, T1 in groups S and M, and in D, T2—group L. Another gene, HORVU6Hr1G080670, encoding bZIP transcription factor, was downregulated only in H (T1, group M). Eight of the detected DEGs were annotated to dehydrin (various types), and almost all of them were overexpressed in response to drought (D, HD) in T2 across flag leaf size groups. Significantly, they were also upregulated in T1 in group M. Gene HORVU5Hr1G092100 (dehydrin DHN2) was overexpressed only in group M in D, T2. Similarly, HORVU4Hr1G074130 encoding annexin D4-like was exclusively overexpressed in group M in T2 (D and HD). In contrast, another gene encoding annexin (D1), HORVU7Hr1G037080, had increased expression in groups S and L (but not in group M) in T2 in HD and D, respectively. Both genes were also annotated for heat response.

##### DEGs assigned to heat response

No GO term annotated to “response to heat” was found on the base of calculated marginal distribution of DEGs (**Figure 3**). Thus, to gain an overview of DEGs related to heat, we analyzed the regulation status of genes present in our dataset annotated as follows: “response to heat” (GO:0009408), “cellular response to heat” (GO:0034605), “regulation of cellular response to heat” (GO:1900034), “heat shock protein binding” (GO:0031072), “heat acclimation,” and “positive regulation of transcription from RNA polymerase II promoter in response to heat stress” (GO:0061408) (**Supplementary Table 6**). This revealed 32 DEGs and nine of them were repeated within mentioned GO categories. The most numerous were genes encoding heat shock proteins (HSPs). Regulation status of heat-related DEGs was not uniform. Eight genes were downregulated and the most DE events were observed in group M in T2, mainly in H and/or HD. Four genes were shared between these treatments in group M over time. They encoded HSP23 (HORVU2Hr1G077710, HORVU6Hr1G077710), HSP70 (HORVU3Hr1G086500), and HSF (HORVU7Hr1G087690). In the same contrasts, gene HORVU5Hr1G094380 (putative HSF-type), was specifically underexpressed in response to H. In contrary, there were 11 upregulated genes, mainly in T2 in groups S and M in D, HD. However, HORVU4Hr1G063350 (HSP20-like chaperone) had increased expression only in T1 in group M across treatments. The expression of another gene encoding HSP20 (HORVU0Hr1G020420) was additionally increased in HD in group S and L (also in H in T2) in both time points. Interestingly, gene HORVU3Hr1G069590 encoding heat shock factor C1b showed enhanced expression always in response to D and HD in T2 in all groups of flag leaf size; it was also confirmed for group M in T1. In turn, gene HORVU7Hr1G088920 encoding heat shock factor C2b was upregulated specifically to group M in D and HD, T2, similarly to gene HORVU4Hr1G074130 encoding annexin D4-like as mentioned previously. Noteworthy, 13 DEGs within group M were found to have opposite regulation status dependent on time or stress treatment. They were upregulated in T1 mostly in D and/or HD, and not changed significantly in H (with two exceptions), whereas in T2 all of them were downregulated in H, and interestingly, they became not significant in D (with two exceptions). One of such gene, HORVU4Hr1G090090 encoding heat-responsive transcription factor HSF85, was upregulated in D and HD but downregulated in H. Two genes encoding HSP20 (HORVU3Hr1G020500, HORVU3Hr1G020490) changed expression status also in response to HD, from positive in T1 to negative in T2. All significant DE events observed for groups S and L showed “upregulation” status.

##### DEGs assigned to abscisic acid

Sixteen DEGs annotated to “response to abscisic acid” were found whose expression was upregulated in reaction to stress factors with one exception, i.e., the drought-related gene HORVU7Hr1G085130 described above (**Figure 3**, **Supplementary Table 5**, **Supplementary Table 6**). In fact, most of the detected DEGs encoded dehydrins, and they were also assigned to drought responses, so the same behavior was observed. In turn, gene HORVU3Hr1G069590 (encoding HSFC1b) was annotated also to heat response. Additionally, we identified HORVU1Hr1G059950 (encoding Em protein CS41) as overexpressed across all groups defined by flag leaf size, mainly in D at both time points. Otherwise, HORVU6Hr1G058000 encoding ECERIFERUM 1-like had increased expression only in group S (D and HD, T2), whereas HORVU7Hr1G045630 encoding protein HVA22, was upregulated in groups M and L in D, HD in T2.

In contrast, there were few downregulated DEGs assigned to the GO term “abscisic acid-activated signaling pathway,” including genes encoding the bZIP transcription factor and the abscisic acid receptor PYL2 mentioned above. Another gene, HORVU4Hr1G055220, encoding the abscisic acid receptor PYL4, had reduced expression in group S in D, HD in T2, and in group M in D over time (**Supplementary Table 6**).

Indeed, we found 19 additional DEGs encoding LEA proteins, based on the Interpro database, but they were not annotated to the ABA response (**Supplementary Table 1**). In general, they belonged to LEA1 and LEA2 subgroups and were overexpressed in groups S and M in response to D and HD in T2. However, two genes were downregulated in HD in T2 across groups of flag leaf size (except for group M for HORVU4Hr1G026770), and a third gene, HORVU7Hr1G012310 (encoding LEA1), had reduced expression only in group S in D, HD in T2. None of these genes were significantly affected by H.

##### DEGs assigned to lipid transport

Curiously, we identified the term “lipid transport” within GO categories with fully upregulated DEGs (**Figure 3**, **Supplementary Table 5**). All of them (with one exception) belonged to the family of lipid transfer protein (LTP) encoding genes, including ns-LTP6, the ns-LTP1 precursor, and three ns-LTP2-like genes. They were upregulated mainly in response to D and HD in groups S and M; however, HORVU5Hr1G109100 was exclusively overexpressed in H (group S in T2, group M in T1) (**Supplementary Table 6**).

##### DEGs assigned to leaf development

Due to the research on genotypes with different flag leaf sizes in our experiment, we decided to follow the reaction of DEGs assigned to the term “regulation of leaf development” (GO:2000024). Only one such DEG, HORVU1Hr1G046740, was found (**Supplementary Table 6**). It encoded the YABBY protein and showed reduced expression induced by individual drought in groups S and L in T2. No expression change was observed in group M. To expand the panel of genes involved in leaf development, we used the Plant Reactome database (**Supplementary Table 1**). This revealed 10 additional DEGs associated with the “regulation of leaf development” pathway, including six genes encoding enzymes. Four of them encoded cysteine protease, and in three cases the expression was increased in D and HD in T2 across all groups defined by flag leaf size; it was also confirmed in T1 in group M. Interestingly, a fourth gene, HORVU7Hr1G119930, was downregulated specifically in group S in D, HD in T2. Similar expression regulation was observed for two genes encoding cytochrome P450, i.e., HORVU5Hr1G057180 and HORVU5Hr1G081060 (downregulated additionally in group L). We detected one gene, HORVU5Hr1G097900, encoding putative TF RL9 (leaf rolling), which had reduced expression in D and HD in groups S and L in T2. None of the DEGs were significantly affected by H.

### Single nucleotide polymorphism

SNP calling in mRNA-seq data revealed 17,261 polymorphic markers (homozygous), including 16,875 SNPs with some effects on protein translation assigned by the VEP tool (**Table 4**). There were 4,552 genes containing SNPs, whose number in a single gene ranged from 1 to 45 (**Supplementary Table 7**). SNP HIGH impact effects were the most abundant due to the introduction of stop codons (stop_gained_variant), whereas SNP MODIFIER effects were more numerous in 5’ or 3’ UTRs and less in introns or non-coding transcripts.

**Table 4:** Classification of SNPs by the type of variant and type of predicted effect by VEP.

Type of variant	Effects predicted by VEP (Ensembl Plants)				
	HIGH	LOW	MODERATE	MODIFIER	Total
3_prime_UTR	0	0	0	3,131	3,131
5_prime_UTR	0	0	0	1,403	1,403
intron	0	0	0	750	750
missense	0	0	4,506	0	4,506
non_coding_transcript_exon	0	0	0	773	773
splice_acceptor	23	0	0	0	23
splice_donor	28	0	0	0	28
start_lost	31	0	0	0	31
stop_gained	71	0	0	0	71
stop_lost	16	0	0	0	16
stop_retained	0	10	0	0	10
synonymous_variant	0	6133	0	0	6,133
Total	169	6,143	4,506	6,057	16,875

Some association between genetic composition and flag leaf size was confirmed by PCoA analysis, i.e., MCam67 with a large flag leaf was significantly distanced from the other group of genotypes, and MCam48 and MCam109, representing a group of medium flag leaf size, showed the closest similarity (**Supplementary Figure 2**).

Gene Ontology term enrichment analysis of genes with HIGH translation effects revealed overrepresented terms only within the molecular function category, mostly associated with the binding of different compounds; the largest fold enrichment was observed for the term “ABC-type transporter activity” (**Supplementary Table 8**).

Next, we analyzed whether DEGs, containing SNPs with HIGH translation effect and being polymorphic between groups of genotypes defined according to flag leaf size, reacted differentially under stress or not. Among the 12 genes identified, only two seemed to have some relationship between genetic polymorphism and gene expression. Gene HORVU3Hr1G002550 (protein kinase domain) was polymorphic between plants of group M (allele C/C) and groups S and L (allele T/T); it was not differentially expressed in group M in any case, but it was downregulated in HD, T2 in groups S and L (also in D). Similarly, gene HORVU6Hr1G084070 (assigned to response to drought/ABA) showed SNP polymorphism between plants of groups M (allele T/T, with exception of MCam109) and S and L (allele C/C) but the only difference in expression was that in group M the gene was overexpressed in both time points in D, HD, whereas in groups S and L DEG was observed only in T2. Within DEGs assigned to another of selected GO terms mentioned above only one gene, HORVU7Hr1G101310, with SNP of HIGH translation effect was detected in subset annotated to lipid transport. However, despite the SNP polymorphism found between groups L (allele C/C) and S and M (allele G/G), there were no differences in the regulation of gene expression across genotypes.

### Evaluation of phenotypes

The effects of applied stress factors on phenotype were similar in all three groups of genotypes. The mean effect of combined HD on the phenotype was more similar to the effect of D than H. In general, a negative effect of adverse conditions on studied traits was observed, excluding the number of tillers (T1) and basal internode length of the main stem (T5). The largest reduction under HD or D was found for grain-related traits (T15, T16, T19–T21). and the loss (by about max. 80%) was, in general, the greater the smaller the size of the flag leaf (**Supplementary Figure 3A**). Size leaf-dependent differences in phenology were noticeable for mean values of flag leaf and heading stages, i.e., with the increase in flag leaf size, plants reached the phase earlier, even by a dozen days; a slight stress-induced alternation was noted in both stages. On the other hand, D and HD caused the greatest delay in reaching full maturity (approximately 10 days) within group L relative to C and H. ANOVA confirmed the significant (P <0.001) influence of flag leaf size on phenology (T24–T26) (**Supplementary Table 9A**). In turn, a significant effect of treatment (P <0.001) and interaction of flag leaf size × treatment (P <0.01) were observed only for full maturity (T26). An ANOVA revealed a significant effect of all sources of variation in the case of nine post-harvest traits (T3, T4, T8, T9, T13, T14, T19, T20, and T22). Treatment and flag leaf size had strong (P <0.001) impact on most of traits, whereas their interaction affected significantly half of analyzed traits (at least at P <0.01). The greatest stress-induced differentiation of mean values of chlorophyll fluorescence parameters was found for Pi_Abs and DI0_RC being, in general, reduced and increased, respectively, especially in T2 (**Supplementary Figure 3B**). Combined HD caused the strongest reduction of the Pi_Abs in all flag leaf size groups, with the greatest extent (2.5-fold decrease relative to control) in group M. Parameter DI0_RC showed the largest positive reaction (2-fold increase), again in group M under D and HD. Other parameters were slightly affected by adverse conditions, excluding small flag leaf size genotypes exposed to H at the first time point (T1), where the greatest mean values of all parameters were identified (except for DI0_RC). An ANOVA revealed the significant effect of all sources of variation on most of the chlorophyll fluorescence parameters, mainly at P <0.001 (**Supplementary Table 9B**). The largest significant effects, on all traits, were observed for treatment and its interactions with time and flag leaf size (excluding Pi_Abs, significant at P <0.01). On the other hand, flag leaf size had the lowest impact on photosynthetic properties, and only Fv_Fm (P <0.001) and Pi_Abs (P <0.01) were significantly affected. The effect of time and interaction time × flag leaf size was significant for seven parameters.

**Figure 4:**
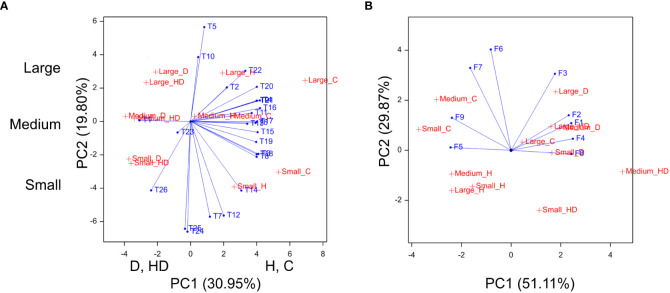
Biplots for phenotypic traits **(A)**, chlorophyll fluorescence parameters at T2 **(B)**. In **(A)**, axis PC1 discriminates treatments C, D, H, and HD, axis PC2—groups of genotypes classified according to the flag leaf size: large, medium, and small; T1–T26, phenotypic traits and phenological stages, F1–F9, chlorophyll fluorescence parameters.

Principal Component Analysis (PCA) confirmed the similar behavior of phenotypic (**Figure 4A**) and chlorophyll fluorescence (**Figure 4B**) traits within the two treatment groups, i.e., a similar effect of conditions C and H as well as a coincidence between D and HD impact.

**Figure 5:**
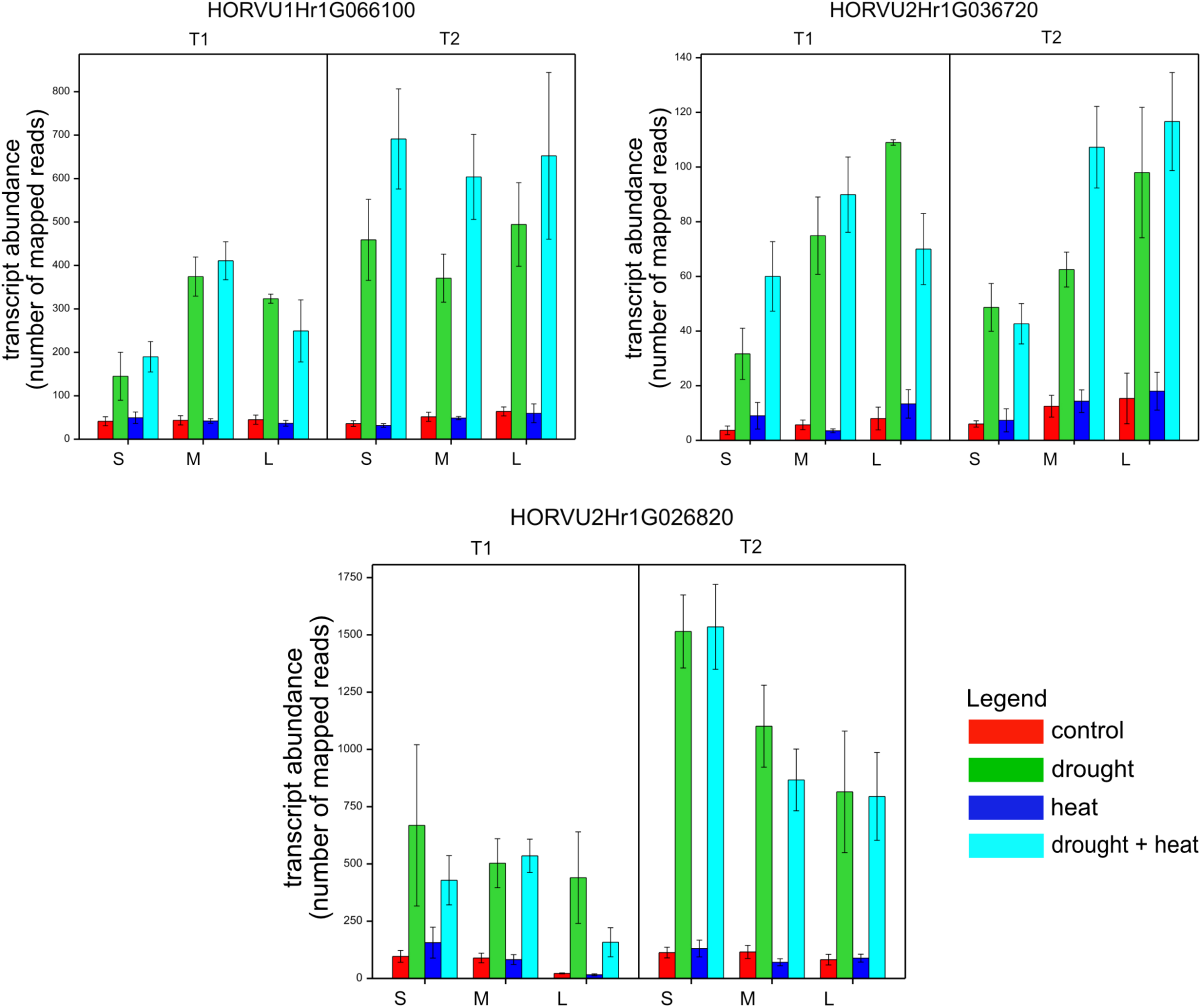
Mean expression levels (counts of mapped reads) for three genes in genotypes of different flag leaf size (S, small; M, medium; L, Large) in two time points (T1 and T2), whose reactions to stresses correlated with reaction with respect to grain weight per plant (T21).

We also conducted a correlation network analysis of phenotypic traits (post-harvest) and gene expression on contrasts between D, H, and HD v. C. This revealed a significant and negative relationship between reactions of expression of three genes, HORVU1Hr1G066100 (r = 0.65), HORVU2Hr1G026820 (r = 0.61), HORVU2Hr1G036720 (r = 0.65), and differences in grain weight per plant (T21), i.e., increased expression of each gene induced under D and HD and reduced grain weight relative to control, observed independently of flag leaf size (**Supplementary Table 1**, **Figure 5**).

## Discussion

High-throughput sequencing of mRNA uncovered drought- and heat-responsive genes whose expression was differentially affected by individual and combined abiotic stress in barley flag leaf. In the global sense, the transcriptomic signature under double stress was more similar to effects caused by drought than by high temperature. Evidently, plants perceived drought as more severe than heat in our experimental setup. It was suggested that under co-occurrence of stressors, plants might prioritize the response strategy to the stress that impacts first or whose effect is perceived more intensely (Kumar Pandey et al., 2015). Prasch and Sonnewald (2015) pointed out four ways of plant response to combined stress: additivity, synergism, idiosyncrasy, and dominance, indicating that idiosyncrasy was the most often reported by researchers in various plant species exposed to multiple stressors. For example, a rather unique molecular response to simultaneous drought and heat compared to individual stresses was observed in *Arabidopsis* (Rasmussen et al., 2013), sorghum (Johnson et al., 2014), and wheat (Rampino et al., 2012). It was inconsistent with our study, where about 3/4 of all DEGs were common between D and HD. Therefore, we assumed that drought dominated during combinatorial stress treatments and the transcriptomic response was majorly shared than unique. This seems understandable as we applied severe drought and a moderate increase in temperature in the experiment. Similarly, Rizhsky et al. (2004) found that the transcriptomic response of *Arabidopsis* to combined drought and heat was more similar to that induced by single drought than heat, where about half of the transcripts were shared between drought and its combination. Concluding, plant responses to multiple stressors consisted of a combination of shared and unique transcriptomic changes, and their proportions depended on various factors like genotype or stress severity and duration.

Furthermore, expression co-reaction network analysis revealed that some DEGs playing various functions (e.g., genes associated with ABA signaling or lipid catabolism) had similar regulation under drought and under its combination with heat; on the other hand, the coincident expression of such DEGs was observed between heat and control conditions. Interestingly, among them, there were DEGs related to oxidoreductases that showed contrasting behavior, i.e., some were overexpressed and others were downregulated under D and HD in relation to H and C. It would seem that oxidoreductase-related genes should be generally upregulated in response to stressors, as indicated by Gharaghanipor et al. (2022), who observed increased abundance of oxidoreductase-coding transcripts in stress-sensitive barley genotypes under salinity. Also, Szurman-Zubrzycka et al. (2021) identified massive upregulation of peroxidases encoding genes in barley roots under low pH. However, in the same study, authors detected both down- and upregulated genes associated with peroxidases in response to aluminum treatment. Such opposite behavior of mentioned genes was also observed in leaves of sugarcane subjected to drought (Contiliani et al., 2022). Contrasting regulation of genes encoding oxidoreductases can be explained by the fact that they constitute a large family of enzymes with complex functions, and apart from defense against stress, they regulate cell homeostasis and plant development (Wang et al., 2018b).

We confirmed that gene expression was time-dependent, i.e., a four-day difference in exposition to adverse conditions had a large impact on transcriptome re-modeling since more DEGs were found on the seventh day compared to the third day of stress. Secondly, it was proved that stress-induced reaction of most of the early-responsive genes was maintained at the later period of plant exposition to stress. We claimed that stress severity was forcefully perceived by plants in the following days, therefore they needed more time for adjustment to adverse conditions. This situation was also observed for an increased temperature effect over time but to a lesser extent relative to other stress treatments, except for genotypes with medium flag leaf size.

Present study proved that stress-induced re-modeling of transcriptome depended also on the size of the flag leaf blade. It is well known that abiotic stresses affect leaves’ morphology and physiology. Leaf rolling and limping as well as a decrease in leaf size/area under drought have been reported in various species (Yang et al., 2021), including flag leaf modifications in wheat (Willick et al., 2018). Heat stress also causes the curling and wilting of leaves (Siddiqui et al., 2015). Hence, transcriptomic changes dependent on flag life size seem to be expected. We demonstrated that prolonged stress induced the most numerous changes in gene expression in genotypes of small size in the flag leaf. Perhaps the small flag leaf perceived the stress as stronger, and enhanced re-modeling of the transcriptome was required to protect leaf vitality under constrained conditions. Generally, the transcriptomic response of plants with small flag leaves was divergent from others with much more downregulated genes, but deeper insight into selected sets of DEGs showed that the reaction of plants in group S was much more like those belonging to group M than to group L. In turn, when considering only the early-responsive genes (T1 time point), most DEGs were specific to genotypes with medium flag leaf size, especially under D and HD. This group consisted of four genotypes, ensuring some genetic variability; thus, such a quick stress-induced reaction in gene expression can be attributed particularly to the medium size of flag leaf less and the genetic background. However, these findings had no significant effect on chlorophyll fluorescence parameters, and according to variance analysis, only two parameters were influenced by flag leaf size, including Fv_Fm. Parallelly, the maximum quantum efficiency of PSII photochemistry seemed to be the most constant parameter across stress treatments lasting for seven days; that can be expected since Fv_Fm was indicated as almost unaffected in barley leaves under early drought stress (Oukarroumet al., 2007). This is in accordance with the findings of Daszkowska-Golec et al. (2017), who did not observe significant changes in most chlorophyll fluorescence parameters in the barley cultivar Sebastian affected by the onset of drought. However, we found that the DI0_RC parameter responded positively, especially under HD in T2 in genotypes of medium flag leaf size. This may suggest that genotypes in group M can dissipate the excess energy more effectively than genotypes in other groups during stress treatment. On the other hand, genotypes in group M showed the strongest reduction of Pi_Abs. This integrative parameter was proved to be very sensitive to early stress scenarios and it corresponds to PSI and PSII efficiency providing quantitative information about plant status under adverse conditions (Strasser et al., 2004). We speculated that applied stresses can impair more strongly the assimilation of CO_2_ in genotypes of group M because its positive correlation with Pi_Abs has been previously documented (e.g., van Heerden et al., 2007). The negative and additive effects of combined drought and heat on photosynthesis in wheat were reported by Perdomo et al. (2015), however they were not clearly evidenced by our evaluation of chlorophyll fluorescence parameters, probably due to the relatively short-term stress application (7 days).

It can be emphasized that some relationship between flag leaf size and phenotypic traits was identified. For example, plants with larger flag leaf size had slightly better grain yield (T21) under optimal conditions, and trait reduction induced by stress treatment (D, HD) was the greater the smaller the flag leaf size. It can be explained by the fact that larger leaves were able to produce, and transport assimilates to spikes more effectively, especially after re-watering and during the grain filling phase. Also, delayed heading was observed in plants with larger flag leaves, perhaps resulting from the plentiful introduction of heading-related alleles from Syrian parents instead of European parents used to develop the studied material (MCam lines), since the CamB1 genotype was earlier than the semi-dwarf genotype Maresi (Mikołajczak et al., 2017). As expected, abiotic stresses had a negative effect on phenotypic traits, mainly D and HD; however, tiller number and basal internode length increased. This agrees with our previous study on the effect on elevated temperature on barley phenotypes (Mikołajczak et al., 2022). Overall, evaluation of phenotypes and chlorophyll fluorescence parameters revealed the coincidence between effect of double stress and single drought as it was observed also at transcriptome level. Interestingly, the association network analysis revealed that increased expression of three genes corresponded significantly to reduced grain yield under drought and combined drought and heat. One of them encodes universal stress protein (USP), which is involved in a wide range of cellular responses and whose protective role against environmental stresses has been suggested (Chi et al., 2019). Loukehaich et al. (2012) reported that *UPS*-overexpressed tomato mutants had significantly increased ABA content. Therefore, it is tempting to speculate that overexpression of this gene in our study may correspond to an increased level of ABA under stress, and its overaccumulation may lead indirectly, e.g., *via* reduced photosynthesis, to reduced grain yield.

We inferred that the transcriptomic response to D and HD was functionally more concentrated than that of H, because in H the lowest number of enriched GO terms was observed. Functionally, the late response differed from the early ones mainly by activation of phosphorylation and dephosphorylation signaling. It plays one of the central roles in a plant’s signalosome, since cascades of protein phosphorylation and dephosphorylation, mediated by kinases and phosphatases, transmit signals and influence gene expression to adapt the plant to stressors (Yang et al., 2021). Apparently, the defense mechanism of studied genotypes against stress was insufficient in the following days of stress, thus plants intensified the signal transduction *via* phosphorylation/dephosphorylation mechanism.

DEGs identified in most contrasts can be considered housekeeping genes required for plant functioning. Especially, genes regulated commonly across stress treatments can be suggested to be involved in the universal stress response, and thus they may constitute a promising target for plant improvement under climate change (Prasch and Sonnewald, 2015). Based on GO enrichment analysis, we found that such genes were broadly associated with stress response, including drought, heat, oxidative stress, and response to reactive oxygen species, as well as with general processes like protein binding, or more specifically tetrapyrrole binding, and gibberellin 20-oxidase activity. These findings suggested that detoxification of reactive oxygen species mediated by tetrapyrroles (Busch and Montgomery, 2015) was the uniform mechanism to mitigate both drought and heat in our study. In turn, it is well documented that gibberellins (GAs) play a prominent role in whole-plant architecture formation, and GA signaling has been suggested to modulate stress tolerance, whereas GA-20-oxidases are crucial enzymes in the regulation of this phytohormone homeostasis (Kuczyńska et al., 2013; Colebrook et al., 2014). Hence it is justified that genes shared across stress treatments led to GA-20-oxidase term overrepresentation; furthermore, the fundamental role of *HvGa20ox2* linked to the *sdw1* locus has been widely discussed, and its paralogues have been identified in barley (Xu et al., 2017), and one was affected by elevated temperature (Mikołajczak et al., 2022). Photosynthesis-related genes were also identified here, as expected, since it is the most basic and critical process in green plants. Overall, the wide functional interpretation of genes affected by abiotic stress confirmed the enrichment of similar categories as for DEGs shared across treatments, and thus we concluded that the transcriptomic response of flag leaves was mainly concentrated on photosynthesis and adjustment to adverse conditions through numerous processes, including ABA signaling and scavenging of free radicals. Despite the large overlap between genes affected by combined stress and a single drought, a set of DEGs was found to be exclusively regulated during stress co-occurrence. Functionally, they were rather not concentrated, but the enrichment analysis suggested that the unique response to combined drought and heat could be partially determined by the specific activity of some enzymes (e.g., oxidoreductase) and transmembrane proteins (e.g., ion channels). Interpreting the set of DEGs assigned to selected functional GO annotations, we found more specific behavior in some genes. Stress-induced modulation of expression of photosynthesis-associated genes occurred more frequently in genotypes with the smallest flag leaf size, and they were majorly late-responsive to drought and combined stress. Several of them had modified expression only under HD. Transcriptomic changes in genotypes with medium flag leaf size were less numerous, but interestingly, they appeared in response to early stress and were maintained for the following days. These findings overlapped the overall conclusion driven from the whole-genome expression analysis, i.e., genotypes of group S perceived stronger the prolonged stress, while genotypes in group M responded quicker and, in that way, extended stress was not so perturbing at the transcriptome level. Noteworthy, part of this reaction of group M was exclusive, i.e., in terms of the oxidative, osmotic, and heat responses.

Most photosynthesis-related genes were downregulated under constrained conditions, but literally, those who had increased expression were of special interest since such genes can be the target of plant stress tolerance improvement. For example, Liang et al. (2019) proved that drought-tolerant mutants of *Arabidopsis* and *Brassica napus* overexpressed photosynthetic genes during exposition to stress. We found some of these DEGs being upregulated under elevated temperature treatment, especially in short-term stress, but note that heat was not as harmful to plants as drought in our study. Interestingly, we found the HORVU2Hr1G090070 gene, annotated to encode a PsbQ-like protein—a component of the oxygen evolving complex (OEC)—to be overexpressed specifically in genotypes of small flag leaf under late drought. OEC is an essential part of photosystem PSII and catalyzes the separation of molecular oxygen from the water; however, the influence of abiotic stress on OCE functioning has not attracted the relevant attention of researchers so far (Gupta, 2020). Daszkowska-Golec et al. (2019) observed decreased expression of genes encoding components of OEC, e.g., PsbO protein (HORVU2HR1G057700), in barley exposed to drought along with reduced photosynthesis efficiency. Hence, we assumed that upregulation of HORVU2Hr1G090070 can affect positively the photosynthetic activity and supposedly its overexpression may compensate partially the plentiful downregulation of photosynthetic-related genes in genotypes of group S under drought. Other genes upregulated under D and HD in genotypes of groups S and M were assigned to chloroplast GO term and encoded high molecular mass early light-inducible protein (ELIP) HV58, malic enzyme or unknow proteins involved in arginine and proline metabolism and HSFA7/HSFA6B-regulatory network-induced by drought and ABA. All of them have been shown to participate in the defense system against stress factors in various species; malic enzyme increases water use efficiency while improving photosynthesis (Sun et al., 2019); ELIP, although less recognized, has been indicated to protect *Arabidopsis* from photooxidative stress (Hutin et al., 2003). On the other hand, the unique overexpression of 9-cis-poxycarotenoid dioxygenase (*NCED*) encoding genes was found in group M (under D and HD), and it is worth mentioning that rice *NCED3*-overexpressing mutants had increased ABA content and enhanced drought and salt tolerance (Huang et al., 2018). These genes are not remarkably studied in barley, and our evidence may bring a new direction in the further improvement of cereals.

Next, we focused on DEGs assigned to drought and the ABA response, whose expression was mostly enhanced under long-term D and HD, independently of flag leaf size. Again, they were early-responsive to stress in genotypes in group M. They were the most numerously represented by dehydrin-encoding genes, which was reasonable due to the well-known multifunctional role of dehydrins in plants’ adjustment to environmental hazards. It has been reported that some dehydrins can be affected by ABA (Yang et al., 2015), so it was justified that DEGs of dehydrins identified in the present study were annotated to both drought- and ABA-related GO terms. Principally, dehydrins are responsible for stabilizing membranes and enzymes under abiotic stress. Dehydrin-overexpressed mutants of *Arabidopsis* showed enhanced tolerance to drought and salinity (Li et al., 2017). Thirteen dehydrin-encoding genes were identified in the barley genome (Tommasini et al., 2008); eight of these genes were found to be differentially expressed in our experiment, and their upregulation is consistent with the findings of Suprunova et al. (2004), who observed an increased abundance of dehydrin transcripts in wild barley exposed to drought. It is worth mentioning that overexpression of several genes encoding dehydrins was also detected in the flag leaf of the drought-tolerant barley cv. Yousef under conditions of water scarcity (Karami et al., 2013). Interestingly, gene HORVU4Hr1G074130 encoding annexin (D4-like) was exclusively upregulated in genotypes of group M under prolonged drought and its combination with heat, whereas HORVU7Hr1G037080 encoding annexin D1 was overexpressed specifically in genotypes of groups S and L. This may indicate that the inaction of the one gene can be compensated by the activity of the other. Drought-induced upregulation of HORVU7Hr1G037080 was also documented in the study of Harb et al. (2020). Annexins are an important component of Ca^2+^ signaling and alleviate oxidative stress. In *Arabidopsis*, enhanced expression of the annexin-1-encoding gene improved drought tolerance (Konopka-Postupolska et al., 2009). We found genotype-independent upregulation of HORVU3Hr1G069590 under drought, being the ortholog of the *EM1* gene of *Arabidopsis*, which was probably activated in an ABA-dependent manner, i.e., transcription factor ABI5 is known to interact with this gene (Carles et al., 2002); however, we did not observe *HvABI5* to be affected by stress treatments. Presumably, an alternative ABA-dependent regulator of the *EM1* gene may exist as suggested in *Arabidopsis* where *AtABI5* was active only in young seedlings (Yoshida et al., 2015). Additionally, post-translational modifications of ABI5 may occur (Zhou et al., 2015). Other known targets of *ABI5* are genes encoding HVA1 and HVA22 proteins of the LEA family, which ensure cell protection from water deprivation (Casaretto and Ho, 2003). Collin et al. (2020) confirmed upregulation of both genes in barley under drought, whereas we found the HVA22-encoding gene to be overexpressed under extended D and HD (excluding genotypes of group S). However, HORVU4Hr1G074710 putatively associated with HVA1—corresponding Interpro identifier (IPR004238) was assigned to HVA1 according to Uniprot database—had also enhanced expression in genotypes of groups S and M (also in T1) under D and HD in T2. This indicated that both genes were rather late-responsive to stress and their functioning could be interchangeable. Overall, consistent with previous reports, we observed several LEA proteins encoding genes to be upregulated under stress treatments, excluding heat. It was not surprising because LEA proteins were reported to be induced mainly by drought, salinity, and freezing (Kamarudin et al., 2019). Unexpectedly, three LEA-associated genes were downregulated, especially under combinatorial stress. Explanation of this phenomenon in the based on literature data remains enigmatic. Perhaps specific LEAs may play a unique role in response to multiple stresses that has not been discovered yet. Interestingly, decreased expression of genes encoding PYL (Pyrabactin Resistance-like) was found. It is the regulatory component of the ABA receptor that is the best characterized ABA perception mechanism in plants (Verma et al., 2019). Particularly interesting behavior of these genes was detected in genotypes of group S, i.e., HORVU7Hr1G088140 encoding PYL2 was induced by short-term HD, whereas HORVU4Hr1G055220 encoding PYL4 was late-responsive to D and HD. In response to abiotic stress, ABA biosynthesis is promoted in order to activate the expression of stress-responsive genes, but the balance of its level is required during plant adaptation to stress since too high an ABA content can have negative consequences, e.g., reduced photosynthesis efficiency (Wang et al., 2018a). We suggested that reduced expression of PYL encoding genes resulted from the need to limit ABA perception to maintain a certain equilibrium of hormone under constrained conditions. Our conclusion is supported by Xu and Zhu (2020), who examined ABA-deficient signaling mutants of *Arabidopsis* and reported that the negative impact of a high level of ABA was mitigated by blocking its signaling.

Enrichment analysis of GO terms revealed a set of heat-associated genes whose expression pattern was ambiguous. Most of them corresponded to heat shock proteins being, in general, upregulated in response to D and HD and not affected by H. Initially HSPs were believed to be stimulated by high temperature, but nowadays they are known to be induced by various stresses including biotic factors (Park and Seo, 2015). Nonetheless, the extraordinary reduction of several HSPs encoding genes was identified in genotypes of group M in T2 under single heat. Also, prolonged double stress caused the inversion of the regulatory status of two genes encoding HSP20, changing it from positive (early stress) to negative. These were unusual findings since HSP-encoding genes are commonly overexpressed under stress conditions; however, very incidental reports also confirmed the negative effect of heat on the regulation of HSP20 (reviewed by Ul Haq et al., 2019). HSPs’ functioning is coordinated by heat shock factors; thus, in our study, downregulation of the above-mentioned genes could be associated with an overlapped decrease in HORVU4Hr1G090090 expression under heat, which encodes HSF (the putative ortholog of *Arabidopsis* HSFC1).

A multifunction of lipids has been documented, including maintenance of cell membrane integrity or signaling in response to stressors (Mamode Cassim et al., 2019), resulting in the predominant overexpression of genes associated with lipid transport proteins (LTPs) under drought and combined stress. Seventy LTPs encoding genes distributed onto all chromosomes were found in barley; such omnipresence confirms their wide role in plant functioning (Zhang et al., 2019). Interestingly, three DEGs of LTP2-like genes were found in our study, and HORVU1Hr1G083170 was also identified by Duo et al. (2021), and it responded to drought, cold, and salinity in roots and in developing grain. In general, the expression of LTP2-encoding genes has been suggested to be specific to the barley aleurone layer (Opsahl-Sorteberg et al., 2004). Hence, our study provided novel data on stress-induced expression of *HvLTP2* in vegetative tissue of barley. Supposedly, heavy stress caused overproduction of LTP2 protein to be transported from the flag leaf to the developing spike, since the flag leaf is an important reservoir of compounds.

During the analysis of DEGs associated with leaf development, a curious behavior of genotypes in groups S and L was observed. Although their flag leaves were morphologically distant, the common regulatory status of three DEGs under prolonged stress was revealed, i.e., the *RL9* gene (the ortholog of *KANADI* in *Arabidopsis*) and genes encoding YABBY and cytochrome P450. All of them were downregulated under D or HD, and in fact, all may correspond to leaf rolling. Apart from known leaf rolling genes (e.g., *RL9*; Yan et al., 2008), the overexpression of the YABBY (key transcription factor affecting leaf blade and floral organ development; Romanova et al., 2021) encoded gene also resulted in leaf curling in *Arabidopsis* (Yang et al., 2019). In turn, cytochromes P450 belong to a large superfamily of enzymes involved in multiple regulatory mechanisms, including phytohormonal signaling, and therefore they influence plant development. Zhang et al. (2021) proved that cytochrome P450 influenced flag leaf shape in transgenic rice. Leaf rolling is a well-known defense phenomenon under drought to limit leaf area and decrease transpiration as a consequence (Kadioglu et al., 2012). Altogether, these results may indicate some disturbances in flag leaf rolling under stress treatments, which seems to be especially relevant for genotypes of group S where stronger perception of stress causes more intense re-modeling of the transcriptome, as we discussed above.

Afterwards, we attempted to deduce whether differential expression of genes can result from SNP polymorphisms between genotypes. We found that about 18% of DEGs contained at least one SNP mutation. Analyzing individual DEGs with SNPs of HIGH translation effect, we were not able to confirm such a relationship; it was incidental. Additionally, according to PCoA analysis on log2FC values for gene expression contrasts and PCoA on SNP markers there was no general coincidence between SNP polymorphism of genotypes and differential expression of genes induced by stress factors. Enrichment of GO terms of genes with SNPs of HIGH translation effects showed that the ABC-type transporter activity differentiated functionally genotypes the most. The ATP-binding cassette (ABC) transporters belong to a large protein family and execute a multitude of biological functions, constituting a fundamental part of the plant regulome (Kang et al., 2011). Despite the importance of ABC transporters, they have not been thoroughly studied in barley so far. Phylogenetic analysis of ABC transporters encoding genes in barley revealed 131 candidate genes, and the stress-induced expression of only several of them was evaluated by Zhang et al. (2020). The expansion of this research area of barley seems inevitable in the near future.

## Conclusions

Genome-wide scale transcriptomics provided a pioneering insight into genes’ behavior in barley flag leaf exposed to drought, elevated temperature, and their combination. Our study demonstrated that, under combined stress, drought was the dominant factor affecting gene expression. It was also confirmed for phenotypic traits and chlorophyll fluorescence parameters. Drought- and heat-responsive genes were identified, including those associated with photosynthesis, abscisic acid signaling, and lipid transport. Interestingly, our study provided novel data on stress-induced expression of *HvLTP2* genes in vegetative tissue of barley. A set of genes annotated to different functions were identified as being shared across stress treatments, e.g., genes encoding LEA proteins, including dehydrins and HSPs. They can determine the universal stress response and thus constitute a promising target for cereal improvement against multiple abiotic stresses. Likewise, regulation of genes involved in signal transduction mediated by phosphorylation and dephosphorylation may be an effective tool for adaptation to prolonged abiotic stress, as indicated in the present study. Genes encoding enzymes, transmembrane proteins, and regulating photosynthetic efficiency that were specifically affected by combined drought and heat can also pave the way for cereals’ improvement against multiple abiotic stresses. Some relationship between flag leaf size and stress response was detected: we assumed that genotypes with a small flag leaf perceived stronger the prolonged stress, while genotypes with medium flag leaf responded quicker (e.g., dehydrin encoding genes), thus probably the extended stress was not so perturbing at transcriptome level; flag leaf-dependent reduction of grain yield confirmed this assumption. Stress-induced genes specific to flag leaf size were also found, e.g., genes encoding the OEC (group S) complex or *HvNCED* (group M). No general coincidence between SNP polymorphism of genotypes and differential expression of genes induced by stress factors was observed.

## Data availability statement

The RNA-seq data presented in the study are deposited in the ArrayExpress repository, accession number E-MTAB-12438.

## Author contributions

KM and AK conceived and designed the study. KM obtained of funding and administrated the project. KM, AK, and MK performed the experiments and collected the data. PK and MN processed the raw data and conducted statistical and bioinformatics analyses. KM interpreted the data, drafted, and revised the manuscript with subsequent critical input and approval by all authors. KM is responsible for the manuscript as a whole.

## References

[B1] Ahmed I. M. Cao F. Zhang M. Chen X. Zhang G. Wu F. (2013). Difference in yield and physiological features in response to drought and salinity combined stress during anthesis in Tibetan wild and cultivated barleys. PLoS One 8, e77869. doi: 10.1371/journal.pone.0077869 24205003 PMC3812012

[B2] Baniwal S. K. Bharti K. Chan K. Y. Fauth M. Ganguli A. Kotak S.. (2004). Heat stress response in plants: a complex game with chaperones and more than twenty heat stress transcription factors. J. Biosci. 29, 471–487. doi: 10.1007/BF02712120 15625403

[B3] Busch A. W. U. Montgomery B. L. (2015). Interdependence of tetrapyrrole metabolism, the generation of oxidative stress and the mitigative oxidative stress response. Redox Biol. 4, 260–271. doi: 10.1016/J.REDOX.2015.01.010 25618582 PMC4315935

[B4] Carles C. Bies-Etheve N. Aspart L. Leon-Kloosterziel K. M. Koornneef M. Echeverria M.. (2002). Regulation of *Arabidopsis thaliana em* genes: role of *ABI5* . Plant J. 30, 373–383. doi: 10.1046/j.1365-313X.2002.01295.x 12000684

[B5] Casaretto J. Ho T. D. (2003). The transcription factors HvABI5 and HvVP1 are required for the abscisic acid induction of gene expression in barley aleurone cells. Plant Cell 15, 271–284. doi: 10.1105/tpc.007096 12509536 PMC143496

[B6] Chang H.-C. Tang Y.-C. Hayer-Hartl M. Hartl F. U. (2007). SnapShot: Molecular chaperones, part I. Cell 128, 212.e1–212.e2. doi: 10.1016/J.CELL.2007.01.001 17990378

[B7] Chi Y. H. Koo S. S. Oh H. T. Lee E. S. Park J. H. Phan K. A. T.. (2019). The physiological functions of universal stress proteins and their molecular mechanism to protect plants from environmental stresses. Front. Plant Sci. 10. doi: 10.3389/fpls.2019.00750 PMC6560075 31231414

[B8] Colebrook E. H. Thomas S. G. Phillips A. L. Hedden P. (2014). The role of gibberellin signalling in plant responses to abiotic stress. J. Exp. Biol. 217, 67–75. doi: 10.1242/jeb.089938 24353205

[B9] Collin A. Daszkowska-Golec A. Kurowska M. Szarejko I. (2020). Barley *ABI5* (*Abscisic acid insensitive 5*) is involved in abscisic acid-dependent drought response. Front. Plant Sci. 11 1138. doi: 10.3389/fpls.2020.01138 32849699 PMC7405899

[B10] Contiliani D. F. de Oliveira Nebó J. F. C. Ribeiro R. V. Andrade L. M. Peixoto Júnior R. F. Lembke C. G.. (2022). Leaf transcriptome profiling of contrasting sugarcane genotypes for drought tolerance under field conditions. Sci. Rep. 12 9153. doi: 10.1038/s41598-022-13158-5 35650424 PMC9160059

[B11] Daszkowska-Golec A. Collin A. Sitko K. Janiak A. Kalaji H. M. Szarejko I. (2019). Genetic and physiological dissection of photosynthesis in barley exposed to drought stress. Int. J. Mol. Sci. 20. doi: 10.3390/ijms20246341 PMC6940956 31888211

[B12] Daszkowska-Golec A. Skubacz A. Marzec M. Slota M. Kurowska M. Gajecka M.. (2017). Mutation in *HvCBP20* (*Cap binding protein 20*) adapts barley to drought stress at phenotypic and transcriptomic levels. Front. Plant Sci. 8. doi: 10.3389/fpls.2017.00942 PMC5454077 28626467

[B13] dos Santos T. B. Ribas A. F. de Souza S. G. H. Budzinski I. G. F. Domingues D. S. (2022). Physiological responses to drought, salinity, and heat stress in plants: A review. Stresses 2, 113–135. doi: 10.3390/stresses2010009

[B14] Duo J. Xiong H. Wu X. Li Y. Si J. Zhang C.. (2021). Genome-wide identification and expression profile under abiotic stress of the barley *non-specific lipid transfer protein* gene family and its qingke orthologues. BMC Genomics 22, 674. doi: 10.1186/s12864-021-07958-8 34544387 PMC8451110

[B15] Fidler J. Zdunek-Zastocka E. Bielawski W. Bielawski W. (2015). Regulation of abscisic acid metabolism in relation to the dormancy and germination of cereal grains. Acta Soc Bot. Pol. 84, 3–11. doi: 10.5586/asbp.2015.004

[B16] Gharaghanipor N. Arzani A. Rahimmalek M. Ravash R. (2022). Physiological and transcriptome indicators of salt tolerance in wild and cultivated barley. Front. Plant Sci. 13 1039. doi: 10.3389/fpls.2022.819282 PMC9047362 35498693

[B17] Gupta R. (2020). The oxygen-evolving complex: A super catalyst for life on earth, in response to abiotic stresses. Plant Signal. Behav. 15, 1824721. doi: 10.1080/15592324.2020.1824721 32970515 PMC7671056

[B18] Gürel F. Öztürk Z. N. Uçarlı C. Rosellini D. (2016). Barley genes as tools to confer abiotic stress tolerance in crops. Front. Plant Sci. 7. doi: 10.3389/fpls.2016.01137 PMC4971604 27536305

[B19] Harb A. Simpson C. Guo W. Govindan G. Kakani V. G. Sunkar R. (2020). The effect of drought on transcriptome and hormonal profiles in barley genotypes with contrasting drought tolerance. Front. Plant Sci. 11 2060. doi: 10.3389/fpls.2020.618491 PMC7786106 33424910

[B20] Henry R. J. (2020). Innovations in plant genetics adapting agriculture to climate change. Curr. Opin. Plant Biol. 56, 168–173. doi: 10.1016/J.PBI.2019.11.004 31836470

[B21] Hosseini S. A. Hajirezaei M. R. Seiler C. Sreenivasulu N. von Wirén N. (2016). A potential role of flag leaf potassium in conferring tolerance to drought-induced leaf senescence in barley. Front. Plant Sci. 7. doi: 10.3389/fpls.2016.00206 PMC4768371 26955376

[B22] Hsu P. Dubeaux G. Takahashi Y. Schroeder J. I. (2021). Signaling mechanisms in abscisic acid-mediated stomatal closure. Plant J. 105, 307–321. doi: 10.1111/tpj.15067 33145840 PMC7902384

[B23] Huang Y. Guo Y. Liu Y. Zhang F. Wang Z. Wang H.. (2018). 9-cis-Epoxycarotenoid dioxygenase 3 regulates plant growth and enhances multi-abiotic stress tolerance in rice. Front. Plant Sci. 9. doi: 10.3389/fpls.2018.00162 PMC5845534 29559982

[B24] Hutin C. Nussaume L. Moise N. Moya I. Kloppstech K. Havaux M. (2003). Early light-induced proteins protect *Arabidopsis* from photooxidative stress. Proc. Natl. Acad. Sci. 100, 4921–4926. doi: 10.1073/pnas.0736939100 12676998 PMC153656

[B25] Intergovernmental Panel on Climate Change (2021). Future global climate: Scenario-based projections and near-term information. in climate change 2021: The physical science basis. contribution of working group I to the sixth assessment report of the intergovernmental panel on climate change. Cambridge Univ. Press, 553–672.

[B26] International Barley Genome Sequencing Consortium Mayer K. F. X. Waugh R. Brown J. W. S. Schulman A. Langridge P.. (2012). A physical, genetic and functional sequence assembly of the barley genome. Nature 491, 711–716. doi: 10.1038/nature11543 23075845

[B27] Janiak A. Kwasniewski M. Sowa M. Kuczyńska A. Mikołajczak K. Ogrodowicz P.. (2019). Insights into barley root transcriptome under mild drought stress with an emphasis on gene expression regulatory mechanisms. Int. J. Mol. Sci. 20. doi: 10.3390/ijms20246139 PMC6940957 31817496

[B28] Jayakodi M. Padmarasu S. Haberer G. Bonthala V. S. Gundlach H. Monat C.. (2020). The barley pan-genome reveals the hidden legacy of mutation breeding. Nature 588, 284–289. doi: 10.1038/s41586-020-2947-8 33239781 PMC7759462

[B29] Johnson S. M. Lim F.-L. Finkler A. Fromm H. Slabas A. R. knight M. R. (2014). Transcriptomic analysis of sorghum bicolor responding to combined heat and drought stress. BMC Genomics 15, 456. doi: 10.1186/1471-2164-15-456 24916767 PMC4070570

[B30] Kadioglu A. Terzi R. Saruhan N. Saglam A. (2012). Current advances in the investigation of leaf rolling caused by biotic and abiotic stress factors. Plant Sci. 182, 42–48. doi: 10.1016/J.PLANTSCI.2011.01.013 22118614

[B31] Kamarudin Z. S. Yusop M. R. Ismail M. R. Tengku Muda Mohamed M. Harun A. R. Yusuff O.. (2019). LEA gene expression assessment in advanced mutant rice genotypes under drought stress. Int. J. Genomics 2019, 8406036. doi: 10.1155/2019/8406036 32083115 PMC7012254

[B32] Kang J. Park J. Choi H. Burla B. Kretzschmar T. Lee Y.. (2011). Plant ABC transporters. Arab. B. 9, e0153. doi: 10.1199/tab.0153 PMC3268509 22303277

[B33] Karami A. Shahbazi M. Niknam V. Shobbar Z. S. Tafreshi R. S. Abedini R.. (2013). Expression analysis of dehydrin multigene family across tolerant and susceptible barley (Hordeum vulgare l.) genotypes in response to terminal drought stress. Acta Physiol. Plant 35, 2289–2297. doi: 10.1007/s11738-013-1266-1

[B34] Kim D. Pertea G. Trapnell C. Pimentel H. Kelley R. Salzberg S. L. (2013). TopHat2: accurate alignment of transcriptomes in the presence of insertions, deletions and gene fusions. Genome Biol. 14, R36. doi: 10.1186/gb-2013-14-4-r36 23618408 PMC4053844

[B35] Konopka-Postupolska D. Clark G. Goch G. Debski J. Floras K. Cantero A.. (2009). The role of annexin 1 in drought stress in *Arabidopsis* . Plant Physiol. 150, 1394–1410. doi: 10.1104/pp.109.135228 19482919 PMC2705051

[B36] Kosová K. Vitámvás P. Prášil I. T. (2014). Wheat and barley dehydrins under cold, drought, and salinity - what can LEA-II proteins tell us about plant stress response? Front. Plant Sci. 5. doi: 10.3389/fpls.2014.00343 PMC4089117 25071816

[B37] Kuczyńska A. Cardenia V. Ogrodowicz P. Kempa M. Rodriguez-Estrada M. T. Mikołajczak K. (2019). Effects of multiple abiotic stresses on lipids and sterols profile in barley leaves (*Hordeum vulgare* l.). Plant Physiol. Biochem. 141, 215–24. doi: 10.1016/j.plaphy.2019.05.033 31181509

[B38] Kuczyńska A. Surma M. Adamski T. Mikołajczak K. Krystkowiak K. Ogrodowicz P. (2013). Effects of the semi-dwarfing *sdw1/denso* gene in barley. J. Appl. Genet. 54, 381–390. doi: 10.1007/s13353-013-0165-x 23975516 PMC3825292

[B39] Kumar Pandey G. Varotto S. Ramegowda V. Pandey P. Senthil-Kumar M. (2015). Shared and unique responses of plants to multiple individual stresses and stress combinations: physiological and molecular mechanisms. Front. Plant Sci. 6. doi: 10.3389/fpls.2015.00723. www.frontiersin.org. PMC4584981 26442037

[B40] Langfelder P. Horvath S. (2008). WGCNA: an r package for weighted correlation network analysis. BMC Bioinf. 9, 559. doi: 10.1186/1471-2105-9-559 PMC2631488 19114008

[B41] Langfelder P. Horvath S. (2012)Fast r functions for robust correlations and hierarchical clustering. Available at: http://www.ncbi.nlm.nih.gov/23050260 (Accessed October 19, 2022). PMC3465711 23050260

[B42] Li H. (2011). A statistical framework for SNP calling, mutation discovery, association mapping and population genetical parameter estimation from sequencing data. Bioinformatics 27, 2987–2993. doi: 10.1093/bioinformatics/btr509 21903627 PMC3198575

[B43] Liang Y. Kang K. Gan L. Ning S. Xiong J. Song S.. (2019). Drought-responsive genes, late embryogenesis abundant group3 (*LEA3*) and vicinal oxygen chelate, function in lipid accumulation in *Brassica napus* and *Arabidopsis* mainly *via* enhancing photosynthetic efficiency and reducing ROS. Plant Biotechnol. J. 17, 2123–2142. doi: 10.1111/pbi.13127 30972883 PMC6790364

[B44] Liao Y. Smyth G. K. Shi W. (2019). The r package rsubread is easier, faster, cheaper and better for alignment and quantification of RNA sequencing reads. Nucleic Acids Res. 47, e47–e47. doi: 10.1093/nar/gkz114 30783653 PMC6486549

[B45] Li Q. Zhang X. Lv Q. Zhu D. Qiu T. Xu Y.. (2017). Physcomitrella patens dehydrins (PpDHNA and PpDHNC) confer salinity and drought tolerance to transgenic arabidopsis plants. Front. Plant Sci. 8 1316. doi: 10.3389/fpls.2017.01316 28798765 PMC5526925

[B46] Loukehaich R. Wang T. Ouyang B. Ziaf K. Li H. Zhang J.. (2012). SpUSP, an annexin-interacting universal stress protein, enhances drought tolerance in tomato. J. Exp. Bot. 63, 5593–5606. doi: 10.1093/jxb/ers220 22915741 PMC3444279

[B47] Love M. I. Huber W. Anders S. (2014). Moderated estimation of fold change and dispersion for RNA-seq data with DESeq2. Genome Biol. 15, 550. doi: 10.1186/s13059-014-0550-8 25516281 PMC4302049

[B48] Ma Y. Cao J. He J. Chen Q. Li X. Yang Y. (2018). Molecular mechanism for the regulation of ABA homeostasis during plant development and stress responses. Int. J. Mol. Sci. 19. doi: 10.3390/ijms19113643 PMC6274696 30463231

[B49] Mamode Cassim A. Gouguet P. Gronnier J. Laurent N. Germain V. Grison M.. (2019). Plant lipids: Key players of plasma membrane organization and function. Prog. Lipid Res. 73, 1–27. doi: 10.1016/j.plipres.2018.11.002 30465788

[B50] Mareri L. Parrotta L. Cai G. (2022). Environmental stress and plants. Int. J. Mol. Sci. 23 5416. doi: 10.3390/ijms23105416 35628224 PMC9141089

[B51] Mascher M. Gundlach H. Himmelbach A. Beier S. Twardziok S. O. Wicker T.. (2017). A chromosome conformation capture ordered sequence of the barley genome. Nature 544, 427–433. doi: 10.1038/nature22043 28447635

[B52] Mascher M. Wicker T. Jenkins J. Plott C. Lux T. Koh C. S.. (2021). Long-read sequence assembly: a technical evaluation in barley. Plant Cell 33, 1888–1906. doi: 10.1093/plcell/koab077 33710295 PMC8290290

[B53] McLaren W. Pritchard B. Rios D. Chen Y. Flicek P. Cunningham F. (2010). Deriving the consequences of genomic variants with the ensembl API and SNP effect predictor. Bioinformatics 26, 2069–2070. doi: 10.1093/bioinformatics/btq330 20562413 PMC2916720

[B54] Mikołajczak K. Kuczyńska A. Krajewski P. Sawikowska A. Surma M. Ogrodowicz P.. (2017). Quantitative trait loci for plant height in maresi × CamB barley population and their associations with yield-related traits under different water regimes. J. Appl. Genet. 58, 23–35. doi: 10.1007/s13353-016-0358-1 PMC5243891 27447461

[B55] Mikołajczak K. Kuczyńska A. Ogrodowicz P. Kiełbowicz-Matuk A. Ćwiek-Kupczyńska H. Daszkowska-Golec A.. (2022). High-throughput sequencing data revealed genotype-specific changes evoked by heat stress in crown tissue of barley *sdw1* near-isogenic lines. BMC Genomics 23, 177. doi: 10.1186/s12864-022-08410-1 35246029 PMC8897901

[B56] Mikołajczak K. Ogrodowicz P. Ćwiek-Kupczyńska H. Weigelt-Fischer K. Mothukuri S. R. Junker A.. (2020). Image phenotyping of spring barley (*Hordeum vulgare* l.) RIL population under drought: Selection of traits and biological interpretation. Front. Plant Sci. 11. doi: 10.3389/fpls.2020.00743 PMC7296146 32582262

[B57] Mikołajczak K. Ogrodowicz P. Gudyś K. Krystkowiak K. Sawikowska A. Frohmberg W.. (2016). Quantitative trait loci for yield and yield-related traits in spring barley populations derived from crosses between European and Syrian cultivars. PLoS One 11. doi: 10.1371/journal.pone.0155938 PMC4881963 27227880

[B58] Mishra S. K. Poonia A. K. Chaudhary R. Baranwal V. K. Arora D. Kumar R.. (2020). Genome-wide identification, phylogeny and expression analysis of *HSF* gene family in barley during abiotic stress response and reproductive development. Plant Gene 23, 100231. doi: 10.1016/J.PLGENE.2020.100231

[B59] Monat C. Padmarasu S. Lux T. Wicker T. Gundlach H. Himmelbach A.. (2019). TRITEX: chromosome-scale sequence assembly of *Triticeae* genomes with open-source tools. Genome Biol. 20, 284. doi: 10.1186/s13059-019-1899-5 31849336 PMC6918601

[B60] Niu Y. Chen T. Zheng Z. Zhao C. Liu C. Jia J.. (2022). A new major QTL for flag leaf thickness in barley (*Hordeum vulgare* l.). BMC Plant Biol. 22, 305. doi: 10.1186/s12870-022-03694-7 35751018 PMC9229122

[B61] Ojanpera K. Sutinen S. Pleijel H. Sellden G. (1992). Exposure of spring wheat, *Triticum aestivum* l., cv. drabant, to different concentrations of ozone in open-top chambers: effects on the ultrastructure of flag leaf cells. New Phytol. 120, 39–48. doi: 10.1111/j.1469-8137.1992.tb01056.x

[B62] Opsahl-Sorteberg H.-G. Divon H. H. Nielsen P. S. Kalla R. Hammond-Kosack M. Shimamoto K.. (2004). Identification of a 49-bp fragment of the *HvLTP2* promoter directing aleurone cell specific expression. Gene 341, 49–58. doi: 10.1016/J.GENE.2004.06.059 15474287

[B63] Oukarroum A. Madidi S. Schansker G. Strasser R. J. (2007). Probing the responses of barley cultivars (*Hordeum vulgare* l.) by chlorophyll a fluorescence OLKJIP under drought stress and re-watering. Environ. Exp. Bot. 60, 438–446. doi: 10.1016/J.ENVEXPBOT.2007.01.002

[B64] Park C.-J. Seo Y.-S. (2015). Heat shock proteins: A review of the molecular chaperones for plant immunity. Plant Pathol. J. 31, 323–333. doi: 10.5423/PPJ.RW.08.2015.0150 26676169 PMC4677741

[B65] Perdomo J. A. Conesa M.À. Medrano H. Ribas-Carbó M. Galmés J. (2015). Effects of long-term individual and combined water and temperature stress on the growth of rice, wheat and maize: relationship with morphological and physiological acclimation. Physiol. Plant 155, 149–165. doi: 10.1111/ppl.12303 25348109

[B66] Prasad P. V. V. Pisipati S. R. Momčilović I. Ristic Z. (2011). Independent and combined effects of high temperature and drought stress during grain filling on plant yield and chloroplast EF-tu expression in spring wheat. J. Agron. Crop Sci. 197, 430–441. doi: 10.1111/j.1439-037X.2011.00477.x

[B67] Prasch C. M. Sonnewald U. (2015). Signaling events in plants: Stress factors in combination change the picture. Environ. Exp. Bot. 114, 4–14. doi: 10.1016/J.ENVEXPBOT.2014.06.020

[B68] Rampino P. Mita G. Fasano P. Borrelli G. M. Aprile A. Dalessandro G.. (2012). Novel durum wheat genes up-regulated in response to a combination of heat and drought stress. Plant Physiol. Biochem. 56, 72–78. doi: 10.1016/J.PLAPHY.2012.04.006 22609457

[B69] Rapazote-Flores P. Bayer M. Milne L. Mayer C.-D. Fuller J. Guo W.. (2019). BaRTv1.0: an improved barley reference transcript dataset to determine accurate changes in the barley transcriptome using RNA-seq. BMC Genomics 20, 968. doi: 10.1186/s12864-019-6243-7 31829136 PMC6907147

[B70] Rasmussen S. Barah P. Suarez-Rodriguez M. C. Bressendorff S. Friis P. Costantino P.. (2013). Transcriptome responses to combinations of stresses in *Arabidopsis* . Plant Physiol. 161, 1783–1794. doi: 10.1104/pp.112.210773 23447525 PMC3613455

[B71] Reddy P. S. Kavi Kishor P. B. Seiler C. Kuhlmann M. Eschen-Lippold L. Lee J.. (2014). Unraveling regulation of the small heat shock proteins by the heat shock factor *HvHsfB2c* in barley: Its implications in drought stress response and seed development. PloS One 9, e89125. doi: 10.1371/journal.pone.0089125 24594978 PMC3942355

[B72] Rizhsky L. Liang H. Shuman J. Shulaev V. Davletova S. Mittler R. (2004). When defense pathways collide. the response of *Arabidopsis* to a combination of drought and heat stress. Plant Physiol. 134, 1683–1696. doi: 10.1104/pp.103.033431 15047901 PMC419842

[B73] Romanova M. A. Maksimova A. I. Pawlowski K. Voitsekhovskaja O. V. (2021). *YABBY* genes in the development and evolution of land plants. Int. J. Mol. Sci. 22. doi: 10.3390/ijms22084139 PMC8074164 33923657

[B74] Scharf K. D. Berberich T. Ebersberger I. Nover L. (2012). The plant heat stress transcription factor (Hsf) family: Structure, function and evolution. Biochim. Biophys. Acta - Gene Regul. Mech. 1819, 104–119. doi: 10.1016/J.BBAGRM.2011.10.002 22033015

[B75] Schubert M. Lindgreen S. Orlando L. (2016). AdapterRemoval v2: Rapid adapter trimming, identification, and read merging. BMC Res. Notes 9, 88. doi: 10.1186/s13104-016-1900-2 26868221 PMC4751634

[B76] Siddiqui M. H. Al-Khaishany M. Y. Al-Qutami M. A. Al-Whaibi M. H. Grover A. Ali H. M.. (2015). Morphological and physiological characterization of different genotypes of faba bean under heat stress. Saudi J. Biol. Sci. 22, 656–663. doi: 10.1016/J.SJBS.2015.06.002 26288573 PMC4537876

[B77] Strasser R. J. Tsimilli-Michael M. Srivastava A. (2004). “Analysis of the chlorophyll a fluorescence transient,” in Chlorophyll a fluorescence: A signature of photosynthesis. Eds. Papageorgiou G. C. Govindjee (New York: Springer), 321–362. doi: 10.1007/978-1-4020-3218-9_12

[B78] Sun X. Han G. Meng Z. Lin L. Sui N. (2019). Roles of malic enzymes in plant development and stress responses. Plant Signal. Behav. 14, e1644596. doi: 10.1080/15592324.2019.1644596 31322479 PMC6768271

[B79] Suprunova T. Krugman T. Fahima T. Chen G. Shams I. Korol A.. (2004). Differential expression of dehydrin genes in wild barley, *Hordeum spontaneum*, associated with resistance to water deficit. Plant Cell Environ. 27, 1297–1308. doi: 10.1111/j.1365-3040.2004.01237.x

[B80] Suzuki N. Rivero R. M. Shulaev V. Blumwald E. Mittler R. (2014). Abiotic and biotic stress combinations. New Phytol. 203, 32–43. doi: 10.1111/nph.12797 24720847

[B81] Svoboda P. Janská A. Spiwok V. Prášil I. T. Kosová K. Vítámvás P.. (2016). Global scale transcriptional profiling of two contrasting barley genotypes exposed to moderate drought conditions: Contribution of leaves and crowns to water shortage coping strategies. Front. Plant Sci. 7. doi: 10.3389/fpls.2016.01958 PMC5187378 28083001

[B82] Szurman-Zubrzycka M. Chwiałkowska K. Niemira M. Kwaśniewski M. Nawrot M. Gajecka M.. (2021). Aluminum or low pH – which is the bigger enemy of barley? transcriptome analysis of barley root meristem under Al and low pH stress. Front. Genet. 12. doi: 10.3389/fgene.2021.675260 PMC8244595 34220949

[B83] Templer S. E. Ammon A. Pscheidt D. Ciobotea O. Schuy C. McCollum C.. (2017). Metabolite profiling of barley flag leaves under drought and combined heat and drought stress reveals metabolic QTLs for metabolites associated with antioxidant defense. J. Exp. Bot. 68, 1697–1713. doi: 10.1093/jxb/erx038 28338908 PMC5441916

[B84] Tommasini L. Svensson J. T. Rodriguez E. M. Wahid A. Malatrasi M. Kato K.. (2008). Dehydrin gene expression provides an indicator of low temperature and drought stress: transcriptome-based analysis of barley (*Hordeum vulgare* l.). Funct. Integr. Genomics 8, 387–405. doi: 10.1007/s10142-008-0081-z 18512091

[B85] Ul Haq S. Khan A. Ali M. Khattak A. M. Gai W.-X. Zhang H.-X.. (2019). Heat shock proteins: Dynamic biomolecules to counter plant biotic and abiotic stresses. Int. J. Mol. Sci. 20. doi: 10.3390/ijms20215321 PMC6862505 31731530

[B86] van Heerden P. D. R. Swanepoel J. W. Krüger G. H. J. (2007). Modulation of photosynthesis by drought in two desert scrub species exhibiting C3-mode CO2 assimilation. Environ. Exp. Bot. 61, 124–136. doi: 10.1016/J.ENVEXPBOT.2007.05.005

[B87] Verma V. Foulkes M. J. Worland A. J. Sylvester-Bradley R. Caligari P. D. S. Snape J. W. (2004). Mapping quantitative trait loci for flag leaf senescence as a yield determinant in winter wheat under optimal and drought-stressed environments. Euphytica 135, 255–263. doi: 10.1023/B:EUPH.0000013255.31618.14

[B88] Verma R. K. Santosh Kumar V. V. Yadav S. K. Pushkar S. Rao M. V. Chinnusamy V. (2019). Overexpression of ABA receptor *PYL10* gene confers drought and cold tolerance to indica rice. Front. Plant Sci. 10. doi: 10.3389/fpls.2019.01488 PMC6892954 31850010

[B89] VSN International (2017). Genstat for windows 19th edition (UK: VSN Int. Hemel Hempstead).

[B90] Wang M. Lee J. Choi B. Park Y. Sim H.-J. Kim H.. (2018a). Physiological and molecular processes associated with long duration of ABA treatment. Front. Plant Sci. 9. doi: 10.3389/fpls.2018.00176 PMC5826348 29515601

[B91] Wang R.-S. Oldham W. M. Maron B. A. Loscalzo J. (2018b). Systems biology approaches to redox metabolism in stress and disease states. Antioxid. Redox Signal. 29, 953–972. doi: 10.1089/ars.2017.7256 29121773 PMC6104248

[B92] Wehner G. Balko C. Humbeck K. Zyprian E. Ordon F. (2016). Expression profiling of genes involved in drought stress and leaf senescence in juvenile barley. BMC Plant Biol. 16, 3. doi: 10.1186/s12870-015-0701-4 26733420 PMC4702385

[B93] Willick I. R. Lahlali R. Vijayan P. Muir D. Karunakaran C. Tanino K. K. (2018). Wheat flag leaf epicuticular wax morphology and composition in response to moderate drought stress are revealed by SEM, FTIR-ATR and synchrotron X-ray spectroscopy. Physiol. Plant 162, 316–332. doi: 10.1111/ppl.12637 28857201

[B94] Xiao B. Huang Y. Tang N. Xiong L. (2007). Over-expression of a *LEA* gene in rice improves drought resistance under the field conditions. Theor. Appl. Genet. 115, 35–46. doi: 10.1007/s00122-007-0538-9 17426956

[B95] Xu Y. Jia Q. Zhou G. Zhang X.-Q. Angessa T. Broughton S.. (2017). Characterization of the *sdw1* semi-dwarf gene in barley. BMC Plant Biol. 17, 11. doi: 10.1186/s12870-016-0964-4 28086794 PMC5237212

[B96] Xu Y. Zhu Z. (2020). Abscisic acid suppresses thermomorphogenesis in *Arabidopsis thaliana* . Plant Signal. Behav. 15, 1746510. doi: 10.1080/15592324.2020.1746510 32202470 PMC7238878

[B97] Yang X. Lu M. Wang Y. Wang Y. Liu Z. Chen S. (2021). Response mechanism of plants to drought stress. Horticulturae 7, 50. doi: 10.3390/horticulturae7030050

[B98] Yang H. Shi G. Li X. Hu D. Cui Y. Hou J.. (2019). Overexpression of a soybean *YABBY* gene, *GmFILa*, causes leaf curling in *Arabidopsis thaliana* . BMC Plant Biol. 19, 234. doi: 10.1186/s12870-019-1810-2 31159746 PMC6547562

[B99] Yang W. Zhang L. Lv H. Li H. Zhang Y. Xu Y.. (2015). The K-segments of wheat dehydrin WZY2 are essential for its protective functions under temperature stress. Front. Plant Sci. 6. doi: 10.3389/fpls.2015.00406 PMC4467595 26124763

[B100] Yan S. Yan C.-J. Zeng X.-H. Yang Y.-C. Fang Y.-W. Tian C.-Y.. (2008). *Rolled leaf 9*, encoding a GARP protein, regulates the leaf abaxial cell fate in rice. Plant Mol. Biol. 68, 239–250. doi: 10.1007/s11103-008-9365-x 18594992

[B101] Yoshida T. Fujita Y. Maruyama K. Mogami J. Todaka D. Shinozaki K.. (2015). Four *Arabidopsis* AREB/ABF transcription factors function predominantly in gene expression downstream of SnRK2 kinases in abscisic acid signalling in response to osmotic stress. Plant Cell Environ. 38, 35–49. doi: 10.1111/pce.12351 24738645 PMC4302978

[B102] Yu Z. Wang X. Zhang L. (2018). Structural and functional dynamics of dehydrins: A plant protector protein under abiotic stress. Int. J. Mol. Sci. 19. doi: 10.3390/ijms19113420 PMC6275027 30384475

[B103] Zandalinas S. I. Balfagón D. Arbona V. Gómez-Cadenas A. Inupakutika M. A. Mittler R. (2016). ABA is required for the accumulation of APX1 and MBF1c during a combination of water deficit and heat stress. J. Exp. Bot. 67, 5381–5390. doi: 10.1093/jxb/erw299 27497287 PMC5049388

[B104] Zeng X. Bai L. Wei Z. Yuan H. Wang Y. Xu Q.. (2016). Transcriptome analysis revealed the drought-responsive genes in Tibetan hulless barley. BMC Genomics 17, 386. doi: 10.1186/s12864-016-2685-3 27207260 PMC4875595

[B105] Zhang M. Kim Y. Zong J. Lin H. Dievart A. Li H.. (2019). Genome-wide analysis of the barley non-specific lipid transfer protein gene family. Crop J. 7, 65–76. doi: 10.1016/J.CJ.2018.07.009

[B106] Zhang Z. Tong T. Fang Y. Zheng J. Zhang X. Niu C.. (2020). Genome-wide identification of barley *ABC* genes and their expression in response to abiotic stress treatment. Plants (Basel Switzerland) 9. doi: 10.3390/plants9101281 PMC7599588 32998428

[B107] Zhang X. Wang Y. Zhu X. Wang X. Zhu Z. Li Y.. (2021). *Curled flag leaf 2*, encoding a cytochrome P450 protein, regulated by the transcription factor Roc5, influences flag leaf development in rice. Front. Plant Sci. 11 2297. doi: 10.3389/fpls.2020.616977 PMC7907467 33643332

[B108] Zhao J. Lu Z. Wang L. Jin B. (2020). Plant responses to heat stress: Physiology, transcription, noncoding RNAs, and epigenetics. Int. J. Mol. Sci. 22. doi: 10.3390/ijms22010117 PMC7795586 33374376

[B109] Zhou X. Hao H. Zhang Y. Bai Y. Zhu W. Qin Y.. (2015). SOS2-like protein kinase5, an SNF1-related protein kinase3-type protein pinase, is important for abscisic acid responses in *Arabidopsis* through phosphorylation of abscisic acid insensitive5. Plant Physiol. 168, 659–676. doi: 10.1104/pp.114.255455 25858916 PMC4453773

