# Supplementary material for: Transcriptome profiling disclosed the effect of single and combined drought and heat stress on reprogramming of genes expression in barley flag leaf
Source: Front Plant Sci. 2023 Jan 16;13:1096685. doi: 10.3389/fpls.2022.1096685 (PMC9885109; doi:10.3389/fpls.2022.1096685)
Supplement: Supplementary file 2 [file DataSheet_2.pdf]

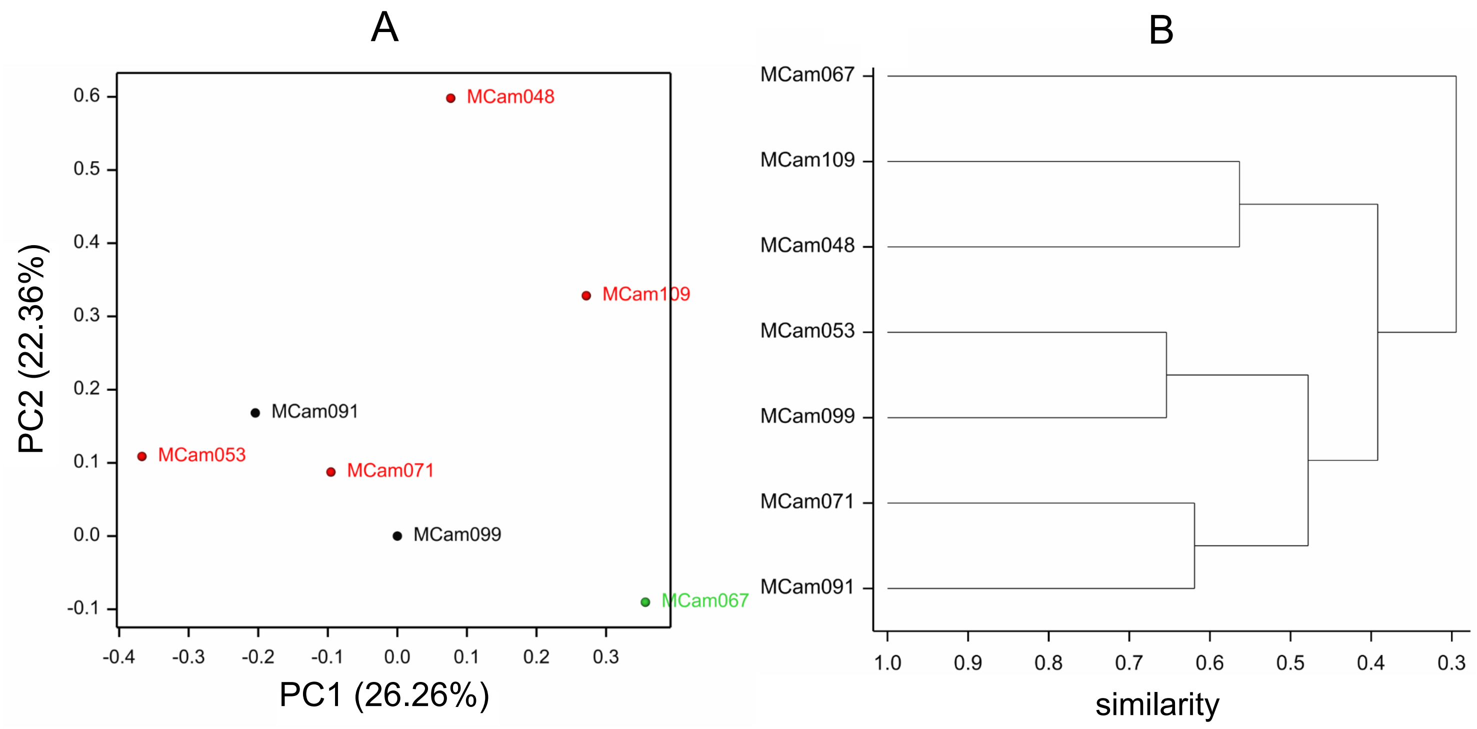

Supplementary Figure 2. Analysis of similarity of genotypes based on SNP data for polymorphisms with HIGH or MODERATE translation effects. **(A)** PCoA results; colors represent genotypes belonging to different flag leaf size groups, *i.e.,* black – small, red – medium, green - large; **(B)** Hierarchical grouping of genotypes. Analyses are based on similarity represented by simple matching coefficients and clustering with the complete link method
