# Supplementary material for: Transcriptome profiling disclosed the effect of single and combined drought and heat stress on reprogramming of genes expression in barley flag leaf
Source: Front Plant Sci. 2023 Jan 16;13:1096685. doi: 10.3389/fpls.2022.1096685 (PMC9885109; doi:10.3389/fpls.2022.1096685)
Supplement: Supplementary file 3 [file DataSheet_3.pdf]

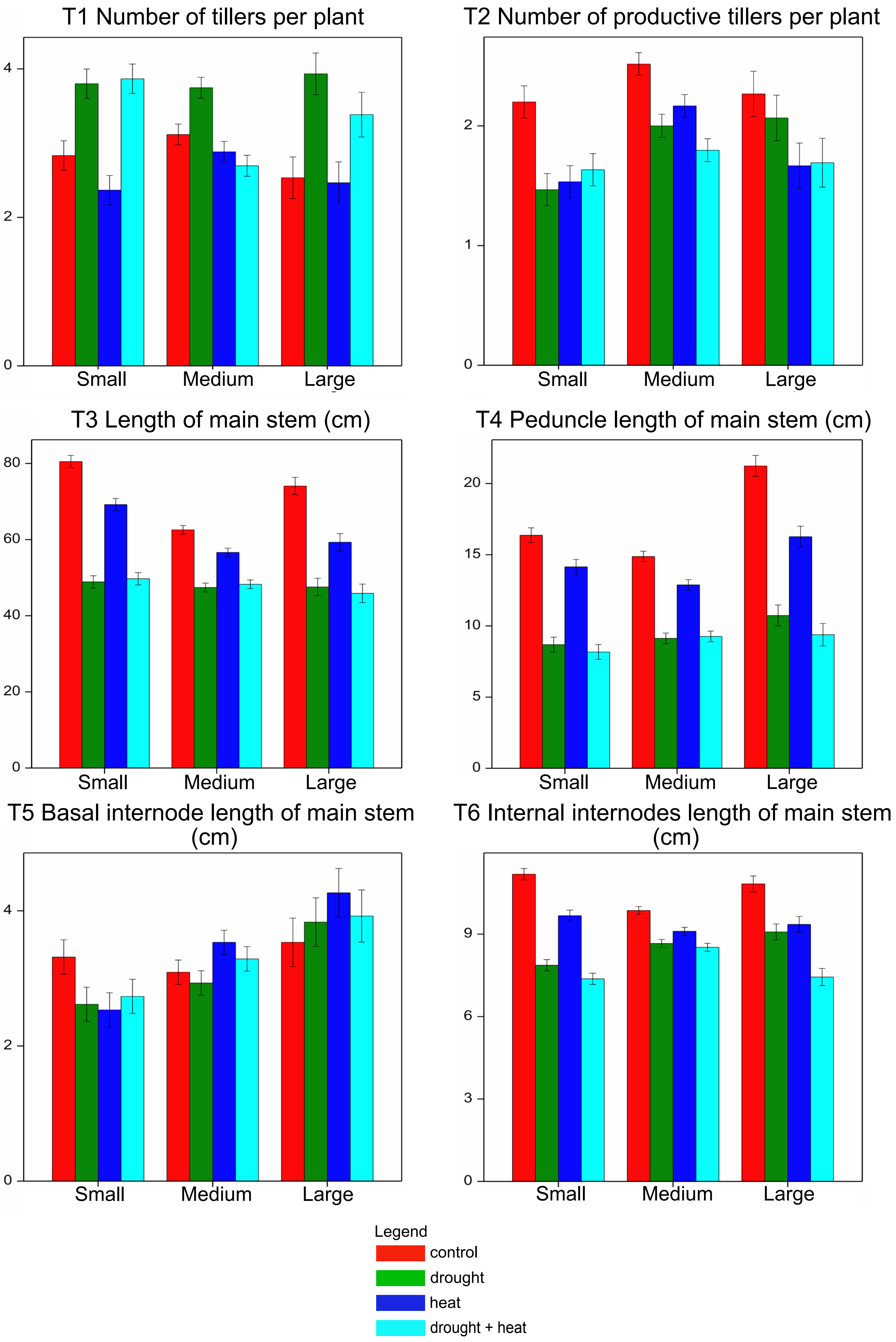

Supplementary Figure 3A. Mean values of phenotypic traits (T1 - T26) calculated for three groups defined by flag leaf size (Small, Medium, Large) across treatments (control, drought, heat, drought and heat)

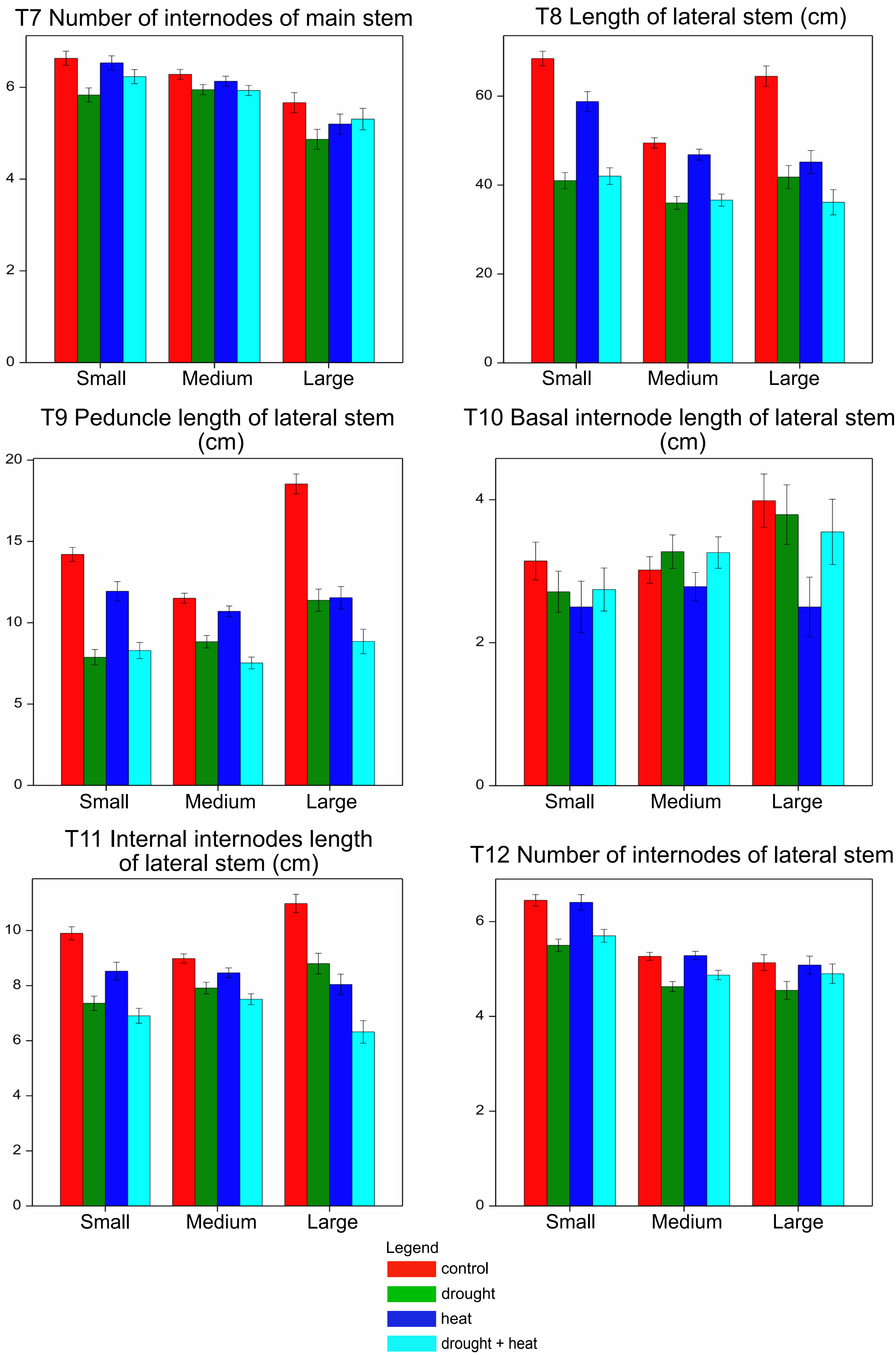

Supplementary Figure 3A. Continued

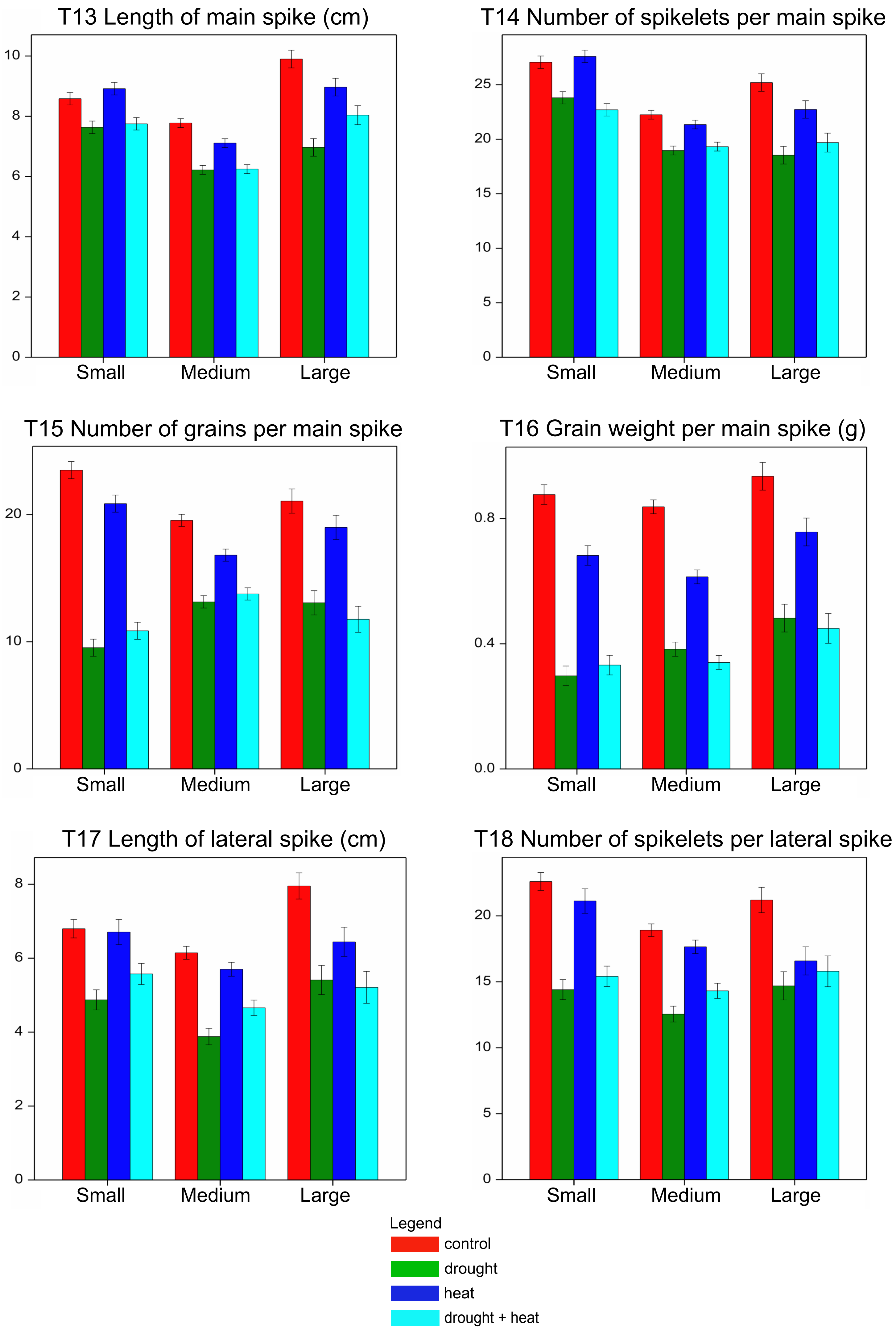

Supplementary Figure 3A. Continued

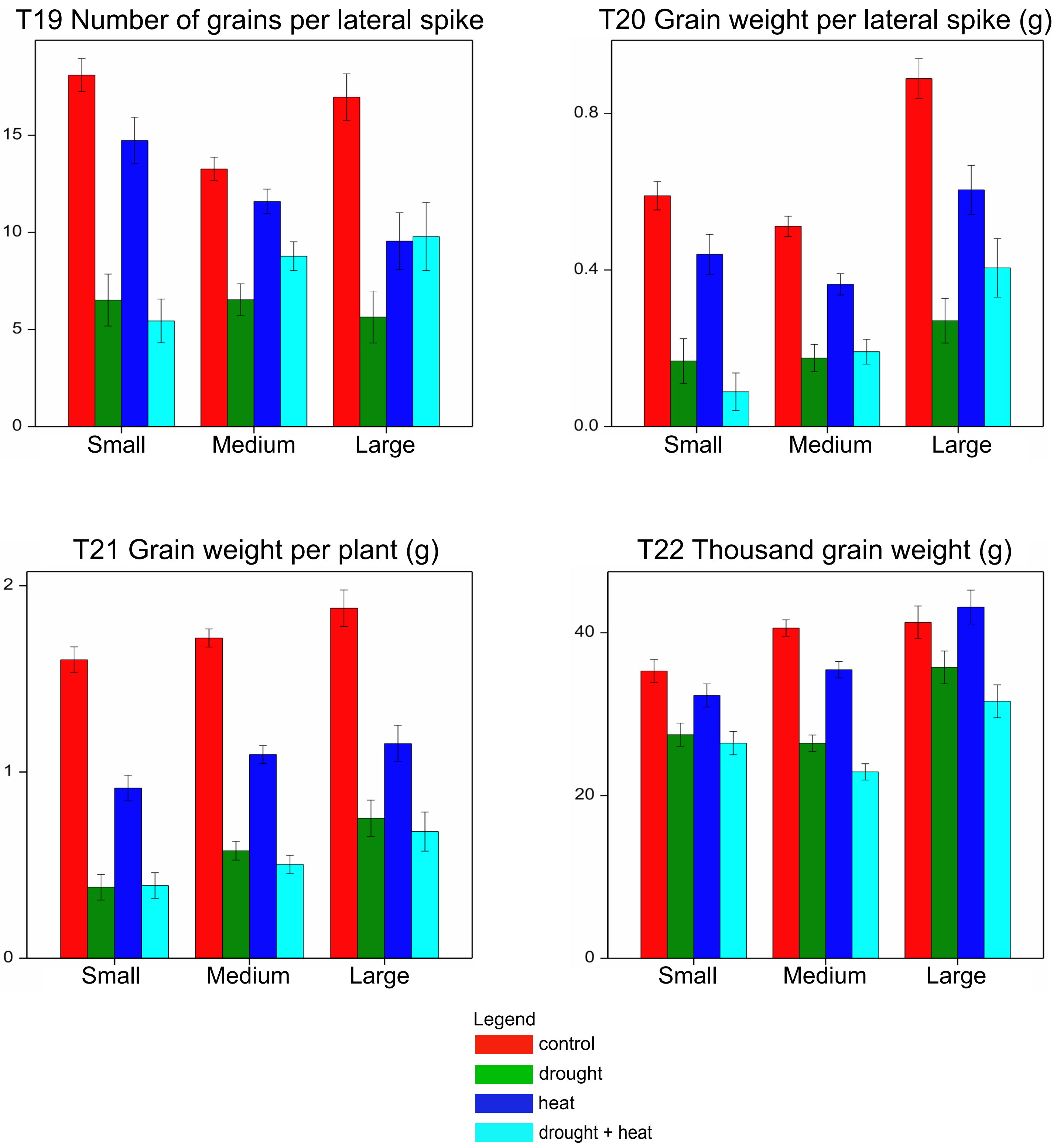

Supplementary Figure 3A. Continued

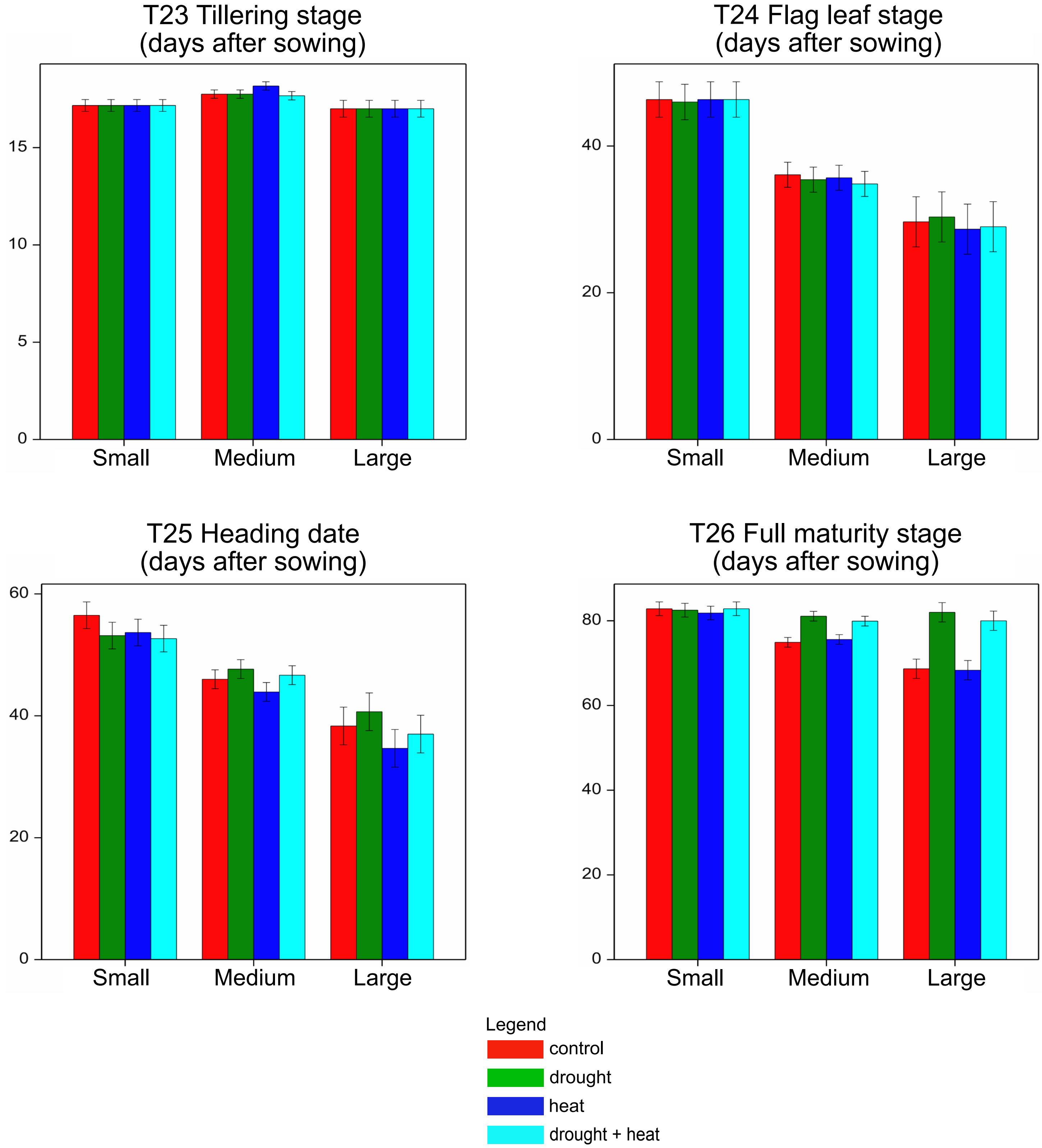

Supplementary Figure 3A. Continued

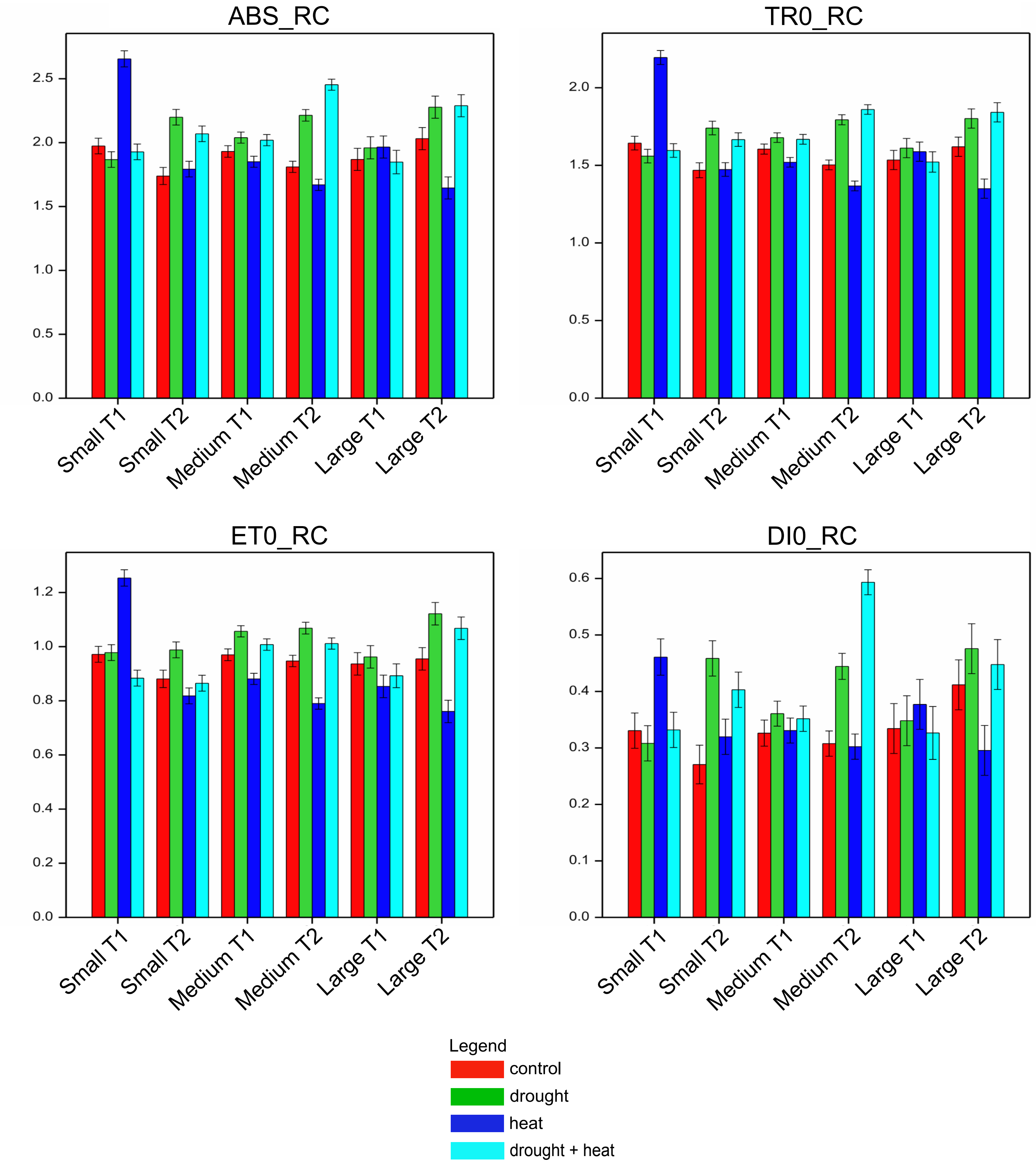

Supplementary Figure 3B. Mean values of chlorophyll fluorescence parameters calculated for three groups defined by flag leaf size (Small, Medium, Large) across treatments (control, drought, heat, drought and heat) in two time points (T1, T2)

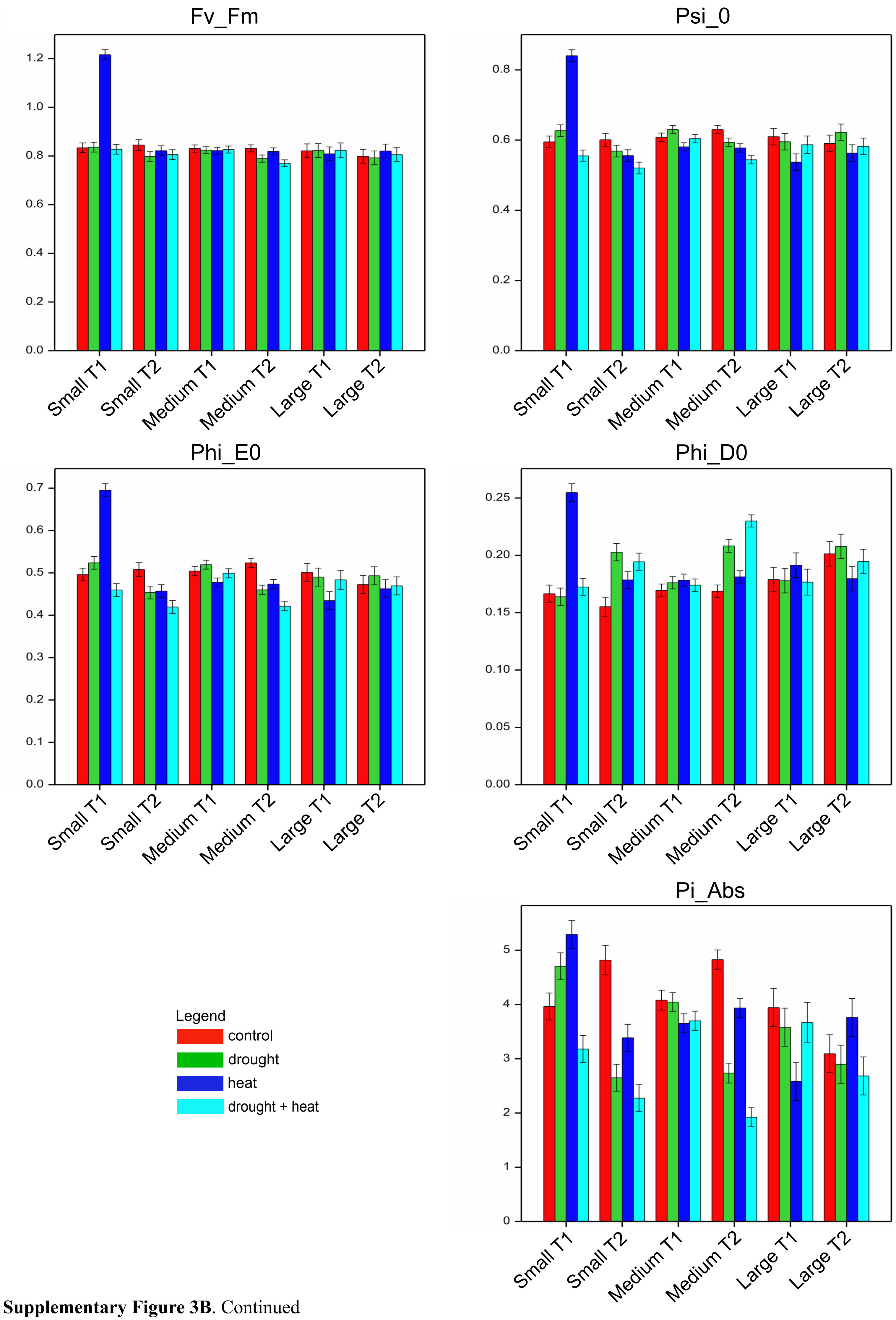

Supplementary Figure 3B. Continued
