# Supplementary material for: Transcriptome profiling disclosed the effect of single and combined drought and heat stress on reprogramming of genes expression in barley flag leaf
Source: Front Plant Sci. 2023 Jan 16;13:1096685. doi: 10.3389/fpls.2022.1096685 (PMC9885109; doi:10.3389/fpls.2022.1096685)
Supplement: Supplementary file 5 [file Table_2.docx]

**Supplementary Table 2**. Overrepresentation analysis of GO Slim terms in set of 1037 DEGs significant in at least three contrasts

(according to geneontology.org tools)

| GO term\|GO identifier | Overrepresentation score (-log_10_(q-value)) for DEGs in groups of contrasts corresponding to | | | | | | | |
| --- | --- | --- | --- | --- | --- | --- | --- | --- |
|  | Flag leaf size groups | | | Treatments | | | Time points | |
|  | S | M | L | D | H | HD | T1 | T2 |
| cellular aromatic compound metabolic process\|GO:0006725 | 1.47 | 2.04 |  |  |  |  |  |  |
| cellular component assembly\|GO:0022607 |  |  |  |  | 3.81 |  |  |  |
| cellular component biogenesis\|GO:0044085 |  |  |  |  | 2.09 |  |  |  |
| cellular localization\|GO:0051641 | 1.91 | 1.36 |  | 1.63 |  | 1.63 | 1.63 | 1.65 |
| cellular nitrogen compound metabolic process\|GO:0034641 | 1.90 | 2.56 |  | 1.52 |  | 1.76 | 1.76 | 1.67 |
| cellular response to oxygen-containing compound\|GO:1901701 | 1.37 | 1.53 |  |  |  |  |  |  |
| defense response\|GO:0006952 | 2.12 | 1.98 |  | 2.04 |  | 2.03 | 2.03 | 1.99 |
| establishment of localization in cell\|GO:0051649 |  |  |  | 1.35 |  |  |  | 1.47 |
| gene expression\|GO:0010467 | 2.30 | 1.99 |  | 1.58 |  | 1.89 | 1.89 | 1.75 |
| generation of precursor metabolites and energy\|GO:0006091 | 2.51 | 2.86 |  | 2.46 | 6.75 | 2.42 | 2.42 | 2.39 |
| heterocycle metabolic process\|GO:0046483 | 1.65 | 2.36 |  |  |  | 1.34 | 1.34 | 1.31 |
| interspecies interaction between organisms\|GO:0044419 | 1.31 |  |  |  |  |  |  |  |
| macromolecule localization\|GO:0033036 | 1.35 |  |  |  |  |  |  |  |
| nucleic acid metabolic process\|GO:0090304 |  | 1.48 |  |  |  |  |  |  |
| nucleobase-containing compound metabolic process\|GO:0006139 | 1.64 | 2.36 |  |  |  |  |  |  |
| organic cyclic compound metabolic process\|GO:1901360 |  | 2.02 |  |  |  |  |  |  |
| organonitrogen compound biosynthetic process\|GO:1901566 |  |  |  | 1.33 |  |  |  | 1.43 |
| photosynthesis\|GO:0015979 | 9.38 | 9.76 |  | 9.64 | 10.00 | 9.43 | 9.43 | 9.51 |
| protein folding\|GO:0006457 |  |  |  |  | 7.66 |  |  |  |
| protein-containing complex assembly\|GO:0065003 |  |  |  |  | 4.87 |  |  |  |
| protein-containing complex subunit organization\|GO:0043933 |  |  |  |  | 4.18 |  |  |  |
| regulation of dephosphorylation\|GO:0035303 | 1.81 | 2.01 |  | 1.75 |  |  |  | 1.73 |
| regulation of phosphatase activity\|GO:0010921 | 1.46 | 1.61 |  | 1.39 |  |  |  | 1.39 |
| regulation of phosphoprotein phosphatase activity\|GO:0043666 | 1.48 | 1.65 |  | 1.43 |  |  |  | 1.43 |
| regulation of protein dephosphorylation\|GO:0035304 | 1.90 | 2.09 |  | 1.84 |  |  |  | 1.81 |
| response to abiotic stimulus\|GO:0009628 | 9.35 | 10.00 |  | 10.00 | 10.00 | 10.00 | 10.00 | 10.00 |
| response to abscisic acid\|GO:0009737 | 6.15 | 6.62 | 4.51 | 6.22 |  | 4.45 | 4.45 | 6.15 |
| response to chemical\|GO:0042221 | 4.04 | 7.53 | 2.58 | 7.20 | 7.89 | 6.13 | 6.13 | 7.06 |
| response to cold\|GO:0009409 | 3.58 | 3.81 | 3.60 | 3.59 |  | 3.49 | 3.49 | 3.56 |
| response to endogenous stimulus\|GO:0009719 | 4.22 | 4.67 | 2.32 | 4.21 |  | 3.03 | 3.03 | 4.13 |
| response to heat\|GO:0009408 |  | 4.22 |  | 4.75 | 10.00 | 4.66 | 4.66 | 4.69 |
| response to hormone\|GO:0009725 | 4.18 | 4.63 | 2.27 | 4.18 |  | 3.00 | 3.00 | 4.10 |
| response to inorganic substance\|GO:0010035 | 4.04 | 9.15 | 3.94 | 9.52 | 9.43 | 9.34 | 9.34 | 9.46 |
| response to light stimulus\|GO:0009416 | 7.35 | 7.92 |  | 7.38 | 9.99 | 7.36 | 7.36 | 7.29 |
| response to lipid\|GO:0033993 | 5.06 | 5.44 | 4.08 | 5.08 |  | 3.50 | 3.50 | 5.02 |
| response to organic substance\|GO:0010033 | 3.43 | 3.81 | 1.77 | 3.30 |  | 2.39 | 2.39 | 3.22 |
| response to osmotic stress\|GO:0006970 |  | 5.31 |  | 6.06 | 10.00 | 6.01 | 6.01 | 6.01 |
| response to oxidative stress\|GO:0006979 |  | 2.86 |  | 3.28 | 9.43 | 3.19 | 3.19 | 3.23 |
| response to oxygen-containing compound\|GO:1901700 | 5.65 | 9.75 | 4.62 | 9.65 | 7.79 | 8.82 | 8.82 | 9.58 |
| response to radiation\|GO:0009314 | 6.97 | 7.55 |  | 7.01 | 9.98 | 6.94 | 6.94 | 6.93 |
| response to stimulus\|GO:0050896 | 7.53 | 9.07 |  | 9.64 | 9.93 | 9.09 | 9.09 | 9.45 |
| response to stress\|GO:0006950 |  | 2.25 |  | 2.97 | 4.86 | 2.93 | 2.93 | 2.88 |
| response to temperature stimulus\|GO:0009266 | 2.52 | 8.56 | 2.65 | 9.00 | 9.99 | 8.91 | 8.91 | 8.92 |
| RNA processing\|GO:0006396 | 1.35 |  |  |  |  |  |  |  |
