# Supplementary material for: Transcriptome profiling disclosed the effect of single and combined drought and heat stress on reprogramming of genes expression in barley flag leaf
Source: Front Plant Sci. 2023 Jan 16;13:1096685. doi: 10.3389/fpls.2022.1096685 (PMC9885109; doi:10.3389/fpls.2022.1096685)
Supplement: Supplementary file 6 [file Table_3.docx]

**Supplementary Table 3**. Normalized mean expression of genes grouped into modules of co-reaction network for studied experimental variants (network constructed for DEGs significant in at least two contrasts).

| Group according to flag leaf size | Time point | Treatment | M1 | M2 | M3 | M4 | M5 | M6 | M7 | M8 | M9 | M10 | M11 | M12 | M13 | M14 | M15 |
| --- | --- | --- | --- | --- | --- | --- | --- | --- | --- | --- | --- | --- | --- | --- | --- | --- | --- |
| S | T1 | D | 0.64 | -0.02 | -0.20 | -0.50 | 0.79 | 0.66 | -0.93 | -0.27 | -0.21 | 0.14 | 0.23 | -0.11 | 0.58 | -0.23 | -0.58 |
| M | T1 | D | 0.04 | 0.51 | 0.91 | -0.21 | 0.01 | -0.06 | 0.59 | 0.24 | 0.11 | 0.07 | 0.67 | -0.04 | 0.30 | 0.51 | 0.09 |
| L | T1 | D | 0.11 | -0.27 | -0.25 | -0.51 | 0.37 | 0.65 | -0.21 | -0.42 | 0.04 | 0.03 | 0.27 | -0.33 | 0.42 | -0.31 | -0.44 |
| S | T2 | D | -1.46 | 1.41 | 1.03 | 1.57 | -1.65 | -2.05 | 1.55 | 1.22 | 1.11 | -0.25 | 1.13 | 0.56 | 1.09 | 1.43 | 1.75 |
| M | T2 | D | -0.25 | 1.71 | 1.61 | 1.58 | -0.45 | -0.11 | 1.55 | 1.80 | 1.38 | 0.08 | 1.18 | 0.35 | 0.96 | 1.33 | 1.80 |
| L | T2 | D | -1.01 | 0.29 | 0.82 | 0.05 | -0.74 | -0.33 | -0.21 | -0.21 | 0.42 | -0.14 | 0.65 | 0.01 | 0.51 | 0.30 | -0.47 |
| S | T1 | H | 0.93 | -0.66 | -1.27 | -0.66 | 0.74 | 0.86 | -1.31 | -0.71 | -0.69 | 0.00 | -0.64 | -0.12 | -0.09 | -0.89 | -0.98 |
| M | T1 | H | 0.96 | -1.12 | -1.09 | -0.54 | 0.82 | 0.31 | -0.25 | -0.92 | -1.19 | 0.15 | -1.46 | -0.07 | -1.81 | -0.80 | -0.59 |
| L | T1 | H | 0.68 | -1.11 | -1.10 | -0.91 | 0.90 | 1.14 | -0.69 | -0.83 | -0.80 | 0.12 | -1.19 | -0.57 | -1.12 | -1.07 | -0.71 |
| S | T2 | H | 1.19 | -1.04 | -0.94 | -0.43 | 0.78 | 0.37 | -0.66 | -0.74 | -0.94 | 0.14 | -1.25 | -0.18 | -1.00 | -0.77 | -0.55 |
| M | T2 | H | 1.03 | -0.98 | -0.75 | -0.52 | 0.96 | 0.83 | -0.59 | -0.99 | -0.83 | -0.02 | -1.23 | -0.44 | -1.39 | -0.91 | -0.50 |
| L | T2 | H | 0.70 | -1.02 | -0.99 | -0.69 | 0.78 | 0.94 | -0.84 | -0.84 | -0.79 | 0.26 | -1.04 | -0.31 | -1.04 | -0.94 | -0.76 |
| S | T1 | HD | 0.17 | -0.30 | -0.62 | -0.62 | 0.29 | 0.21 | -0.99 | -0.36 | -0.46 | -0.26 | -0.05 | -0.07 | 0.38 | -0.46 | -0.57 |
| M | T1 | HD | -0.40 | 0.22 | 0.71 | -0.10 | -0.46 | -0.78 | 0.44 | 0.43 | 0.00 | 0.09 | 0.39 | 0.38 | -0.01 | 0.43 | 0.23 |
| L | T1 | HD | 0.02 | -0.64 | -0.55 | -0.63 | 0.33 | 0.70 | -0.38 | -0.67 | -0.08 | 0.15 | -0.08 | -0.13 | 0.08 | -0.50 | -0.65 |
| S | T2 | HD | -1.57 | 1.15 | 0.86 | 1.40 | -1.96 | -2.13 | 1.32 | 1.33 | 0.94 | -0.37 | 0.88 | 0.47 | 0.87 | 1.26 | 1.32 |
| M | T2 | HD | -0.58 | 1.53 | 1.36 | 1.37 | -0.88 | -1.02 | 1.52 | 1.86 | 1.35 | -0.01 | 1.05 | 0.44 | 0.87 | 1.20 | 1.68 |
| L | T2 | HD | -1.18 | 0.32 | 0.47 | 0.36 | -0.63 | -0.21 | 0.08 | 0.09 | 0.62 | -0.18 | 0.51 | 0.17 | 0.39 | 0.45 | -0.07 |
| Joined module | | | I | II | II | - | I | I | - | - | II | - | III | - | III | II | - |
