# Supplementary material for: Transcriptome profiling disclosed the effect of single and combined drought and heat stress on reprogramming of genes expression in barley flag leaf
Source: Front Plant Sci. 2023 Jan 16;13:1096685. doi: 10.3389/fpls.2022.1096685 (PMC9885109; doi:10.3389/fpls.2022.1096685)
Supplement: Supplementary file 1 [file DataSheet_1.pdf]

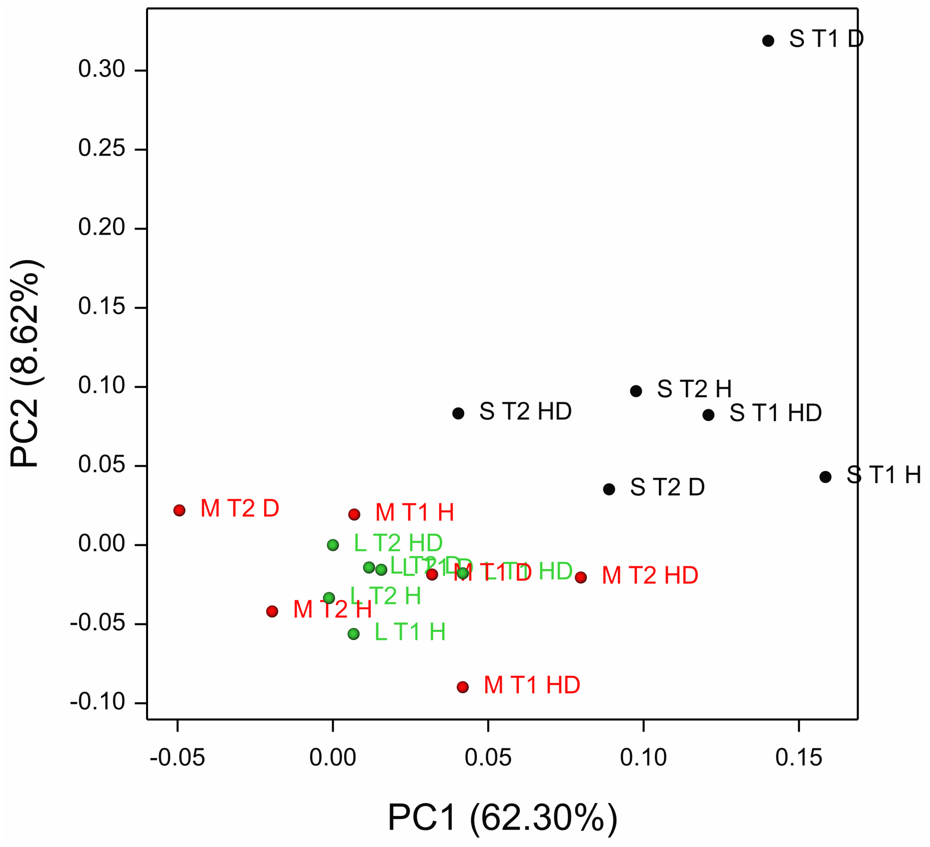


**Supplementary Figure 1**. Principal coordinate analysis on log2FC values for contrasts of genes expression. Points represent profiles of reaction of DEGs under conditions D, H, or HD relative to control, in genotypes with small (S, black), medium (M, red) or large (L, green) flag leaves, at time point T1 or T2
